# Evolutionary Emergence of microRNAs in Human Embryonic Stem Cells

**DOI:** 10.1371/journal.pone.0002820

**Published:** 2008-07-30

**Authors:** Hong Cao, Chao-shun Yang, Tariq M. Rana

**Affiliations:** Department of Biochemistry and Molecular Pharmacology, University of Massachusetts Medical School, Worcester, Massachusetts, United States of America; Katholieke Universiteit Leuven, Belgium

## Abstract

Human embryonic stem (hES) cells have unique abilities to divide indefinitely without differentiating and potential to differentiate into more than 200 cell types. These properties make hES cells an ideal model system for understanding early human development and for regenerative medicine. Molecular mechanisms including cellular signaling and transcriptional regulation play important roles in hES cell differentiation. However, very little information is available on posttranscriptional regulation of hES cell pluripotency, self-renewal, and early decisions about cell fate. microRNAs (miRNAs), 22-nt long non-coding small RNAs found in plants and animals, regulate gene expression by targeting mRNAs for cleavage or translation repression. In hES cells we found that 276 miRNAs were expressed; of these, a set of 30 miRNAs had significantly changed expression during differentiation. Using a representative example, miR-302b, we show that miRNAs in human ES cells assemble into a *bona fide* RISC that contains Ago2 and can specifically cleave perfectly matched target RNA. Our results demonstrate that human ES cell differentiation is accompanied by changes in the expression of a unique set of miRNAs, providing a glimpse of a new molecular circuitry that may regulate early development in humans. Chromosomes 19 and X contained 98 and 40 miRNA genes, respectively, indicating that majority of miRNA genes in hES cells were expressed from these two chromosomes. Strikingly, distribution analysis of miRNA gene loci across six species including dog, rat, mouse, rhesus, chimpanzee, and human showed that miRNA genes encoded in chromosome 19 were drastically increased in chimpanzees and humans while miRNA gene loci on other chrosmomes were decreased as compared with dog, rat, and mouse. Comparative genomic studies showed 99% conservation of chromosome 19 miRNA genes between chimpanzees and humans. Together, these findings reveal the evolutionary emergence, ∼5 million years ago, of miRNAs involved in regulating early human development. One could imagine that this burst of miRNA gene clusters at specific chromosomes was part of an evolutionary event during species divergence.

## Introduction

Embryonic stem (ES) cells are derived from the inner cell mass of the developing blastocyst and have the capacity to divide for indefinite periods without differentiation. Under specific developmental cues and environments, ES cells start to differentiate and can give rise to more than 200 specific cell types that make up an organism [1]. To better understand the biology of embryonic development and to develop the potential of using ES cells in medicine, it is critical to discover the molecular mechanisms that regulate their differentiation and the stages at which cell lineages are induced and specified. Recent developments in isolating human ES cells have facilitated investigations of molecular mechanisms of early human development [2], [3].

The maintenance of ES cells in the undifferentiated state has been shown to depend on 2 transcription factors, OCT4/POU5F1 and NANOG [4]–[6]. OCT4 interacts with the HMG-box transcription factor, SOX2, and regulates gene expression in mouse ES cells [7]–[10]. These 3 transcription factors (OCT4/ POU5F1, SOX2, and NANOG) have also been shown to be essential in the early development and propagation of undifferentiated ES cells (reviewed in [1],[4]–[6],[7]–[10]). Recent genome-scale location analysis of target genes for OCT4, SOX2, and NANOG indicate that these 3 transcription factors collaborate to form autoregulatory and feedforward loops [11]. The OCT4 and NANOG transcription network has also been shown to regulate pluripotency in mouse ES cells [12]. These studies provide molecular understanding of the regulatory circuits involved in the pluripotency and lineage specification of ES cells. However, very little information is available on posttranscriptional mechanisms that regulate ES cell pluripotency, self-renewal, and early decisions about cell fate. Here we show that human ES cell differentiation is accompanied by changes in the expression of a unique set of miRNAs, providing a glimpse of a new posttranscriptional regulatory circuitry that may control early development in humans. Interestingly, the number of hES miRNA genes encoded in chromosome 19 drastically increased in chimpanzees and humans. Comparative genomic studies revealed a high degree of chromosome 19 miRNA conservation between chimpanzees and humans. Our results reveal the evolutionary emergence, ∼5 million years ago, of miRNAs involved in regulating early human development.

## Results and Discussion

MicroRNAs (miRNAs), ∼22-nt long non-coding RNAs, are an abundant class of small regulatory RNAs found in plants and in animals. miRNAs are assembled with Ago2 and other proteins into an effector complex, miRISC, which targets mRNAs for cleavage or translation repression. Thus, miRNAs can play important roles in development by modulating posttranscriptional regulation of target genes [13]–[15]. The human genome encodes hundreds of miRNAs that have the potential to regulate protein expression by thousands of mRNAs [13], [16]. For example, miRNAs modulate differentiation of the hematopoietic lineage [17] and several ES cell-specific miRNA have been identified [18], [19] miRNAs have also been shown to regulate brain morphogenesis in zebrafish [20] and in animal development [21].

**Figure 1:**
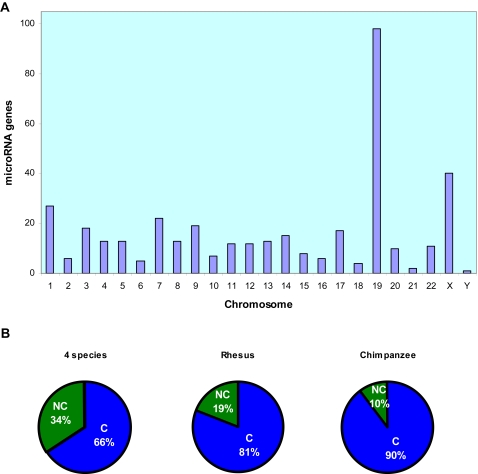
Human embryonic stem cells express 276 miRNAs. (A) 138 hES cell-specific miRNA genes are located on chromosomes 19 and X. Genomic clusters of miRNA were determined by aligning miRNA gene sequences with the human genomic database at Ensemble genome browser by BLAST search (http://www.ensembl.org/Multi/blastview version 41, 2006). Matched chromosome sequences with E values less than 0.01 have been clustered, except specifically marked regions (see Table S1). (B) Evolutionary conservation of hES cell-specific miRNAs. The human genome and genomes of 16 vertebrates (chimpanzee, rhesus monkey, rat, mouse, rabbit, dog, cow, armadillo, elephant, tenrec, opossum, chicken, frog, zebrafish, tetraodon, and fugu) were aligned using the University of California at Santa Cruz Genome Browser (http://genome.ucsc.edu) with the BLAST-Like Alignment Tool (BLAT). C: conserved and NC: nonconserved. Conservation of miRNA genes in 4 species was defined as perfect alignment of at least 3 species among mouse, rat, rabbit, dog and cow (rarely, armadillo, elephant, tenrec or opossum) with a human miRNA sequence. If a miRNA had more than one matched region (repeats) in human chromosomes and only one region was perfectly aligned with other species, it was defined as conserved. Sequence alignments with 2 mismatches or a single mismatch in the miRNA seed region (2–8 nucleotides) were designated as nonconserved (Table S2). miRNA conservation is shown among 4 species, between human and rhesus monkey, and between human and chimpanzee.

We hypothesized that during ES cell differentiation, miRNA expression levels would change to modulate posttranscriptional gene expression, thus providing new insights into the molecular circuitry that controls human ES cell self-renewal and differentiation. To test this hypothesis, small RNAs were isolated from hES cells, and quantification of miRNA expression by miRNA microarray analysis showed expression of 276 miRNAs (Supplementary Table S1). The genomic distribution of these miRNAs was determined by identifying the chromosomal loci of their genes (Table S1), as summarized in Fig 1A. MicroRNA genes were distributed in all chromosomes. However, chromosomes 19 and X contained 98 and 40 miRNA genes, respectively, indicating that majority of the miRNA genes in hES cells were expressed from these two chromosomes.

To investigate the evolutionary emergence of miRNA expression in hES cells, we carried out comparative genomic studies by aligning each gene sequence for the 276 miRNAs with the human genome and the genomes of 16 vertebrates, including mammalian, amphibian, bird, and fish species (Fig 1B and Supplementary Table S2). Drawing from the evolution and time of divergence of various species, we divided the results of our comparative miRNA gene analysis into 3 sections (Fig 1B). First, conservation was determined in 4 species. That is, we analyzed conservation of miRNA gene sequences between human and at least 3 species among mouse, rat, rabbit, dog, or cow (rarely armadillo, elephant, tenrec or opossum); this analysis showed that 66% of miRNA genes were conserved. Second, rhesus monkey and human ES cell miRNA gene-sequence analysis showed 81% conservation. Third, chimpanzee and hES cell miRNA gene-sequence analysis revealed 90% conservation (Fig 1B).

**Figure 2:**
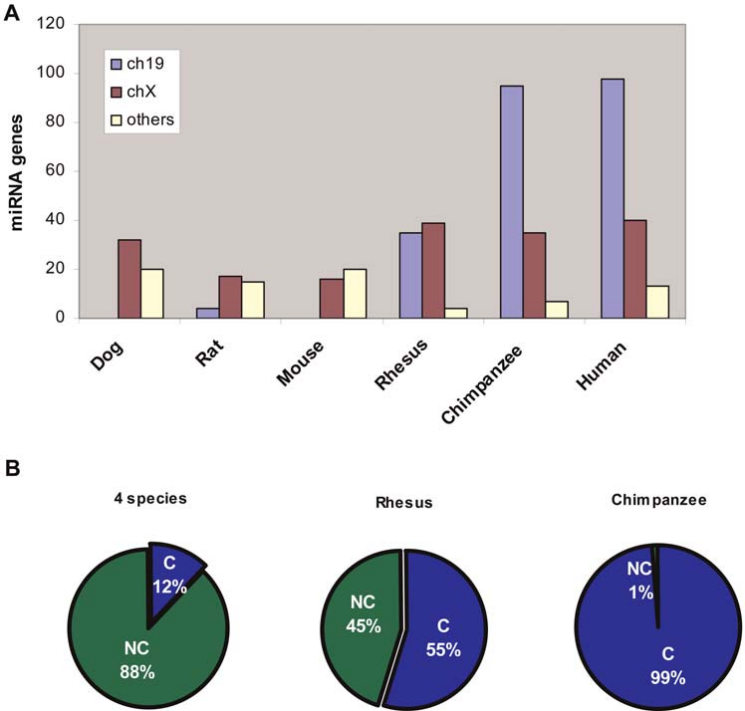
Distribution and conservation analysis of 138 miRNA genes clustered in Chromosome 19 and Chromosome X. (A) miRNA gene distribution across human, chimpanzee, rhesus, mouse, rat and dog. Others: other chromosomes excluded chromosome 19 and chromosome X. (B) Conservations of human chromosome 19 encoded miRNAs: across four species including human, mouse, rat and dog; human and Rhesus; and human and chimpanzee. Conservation of miRNAs was analyzed as described in Fig 1.

The conservation of hES cell miRNA genes among various species suggested a correlation with the evolutionary development time and divergence of species. For example, the evolutionary time for 4 vertebrate species to develop to humans, rhesus to humans, and chimpanzee to humans represents approximately >100, 70, and 5 million years, respectively. Our findings that most of the hES miRNA genes are located on chromosomes X and 19 and that these genes show 90% conservation with a single species, chimpanzee, suggested that the evolution of these miRNAs and their chromosome clustering are recent primate-specific phenomena in the history of evolution. To address this hypothesis, we analyzed the distribution of hES cell specific miRNA gene loci across six species including dog, rat, mouse, rhesus, chimpanzee, and human. This analysis provide an intriguing result indicating that hES cell specific miRNA genes encoded in chromosome 19 was drastically increased in chimpanzees and humans while miRNA gene loci on other chrosmomes were decreased as compared with dog, rat, and mouse (Fig 2A). Similarly, the number of miRNA genes encoded in chromosome X and 19 were increased in rhesus, although to a lesser extent than in chimpanzees and humans. We next determined the conservation of these miRNA genes in various species (Fig 2B) that showed 99% conservation of chromosome 19 miRNA genes between chimpanzees and humans. Taken together, these findings reveal evolutionary emergence of miRNA genes located on chromosome 19. These results also suggest that most of the hES-specific miRNAs evolved ∼5 million years ago.

**Figure 3:**
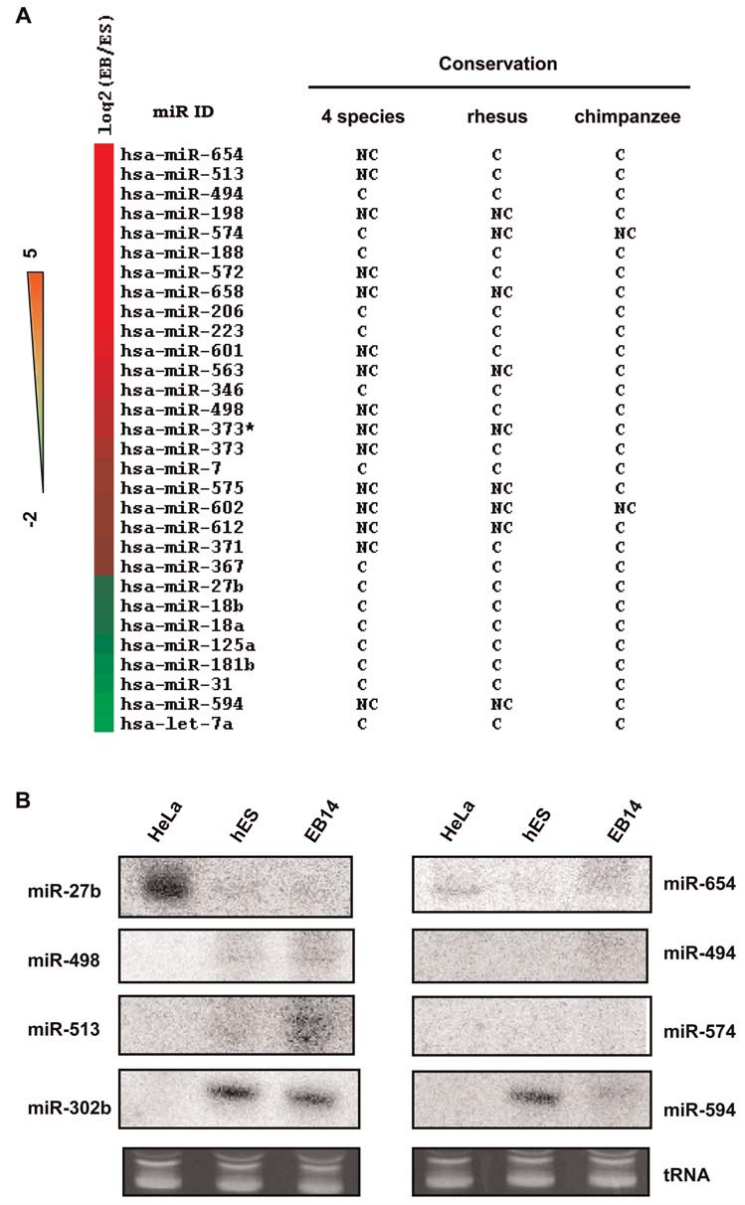
(A) Expression of a unique set of miRNAs changes during differentiation of human ES cells (hES) into embryoid bodies (EBs). miRNA expression was analyzed in hES cells and EBs after 14 days of differentiation. miRNA expression is arranged by log2 ratio in hES cells/EBs (EB/ES) for 30 of ∼276 miRNAs whose log2 ratio >1. Red indicates upregulated miRNAs in EB14 relative to their expression in ES cells and green indicates downregulated miRNAs. Conservation of miRNAs was analyzed as described in Fig 1 (Table S3, supplementary material). (B) Northen analysis of miRNA expression in hES cells and EBs. Total RNA was prepared from hES and 14-days embroid bodies (EB14). RNAs (10 ug) were loaded onto gels and miRNA bands were resolved by 14% denaturing gels. miRNAs were hybridized with various ^32^P labeled specific DNA probes as indicated. Specific bands were visualized by phosphoImager. tRNA band shows the loading control.

If miRNAs are involved in regulating hES cell differentiation by modulating protein expression, then levels of miRNA expression would change when hES cells are induced to differentiate. To address this question, we examined changes in expression of hES cell-specific miRNAs during differentiation. At day 14 after initiating differentiation in hES cells, small RNAs were isolated from hES cells and from embryoid bodies (EBs), which represent hES cell differentiation and form three dimensional colonies, and levels of miRNA expression were quantified by miRNA microarray analysis. Of 276 miRNAs detected, 30 changed significantly, i.e., the log_2_ ratio of miRNA expression in EBs/hES cells (EB/ES) was >1 (Fig 3A and Supplementary Table S3). We found that when hES cells differentiated, 8 miRNAs were downregulated and 22 miRNAs were upregulated. Expression of the most upregulated miRNA, miR-654, changed ∼30-fold during differentiation. Changes in miRNA expression levels and trends were further confirmed by Northern blotting and qPCR analysis. A representative gel shows that hES and EB specific miRNAs were specifically detected in hES and EB14, respectively, and these miRNAs were absent in HeLa cells (Fig 3B). The most downregulated miRNA during EB formation was miR-let-7a, which is of interest because let-7 has been shown to regulate developmental timing in *Caenorhabditis elegans* [22]. Evolutionary conservation analysis of these 30 miRNAs (Fig 3, Supplementary Table S3) revealed that almost all of the downregulated miRNAs were conserved across species, except for miR-594, which was nonconserved in the analysis of conserved miRNA genes between humans and 4 species and between humans and rhesus monkey. Downregulated miRNAs showed complete conservation between humans and chimpanzees. On the other hand, upregulated miRNAs showed a high degree of variation in conservation among species (Fig 3). Most of the miRNAs upregulated during hES differentiation were nonconserved among 4 species (64%) and with rhesus monkey (32%), but were highly conserved with chimpanzee (91%). These results again show a high degree of miRNA conservation between humans and chimpanzees, highlighting the recent evolution of a unique set of miRNAs involved in early human development.

**Figure 4:**
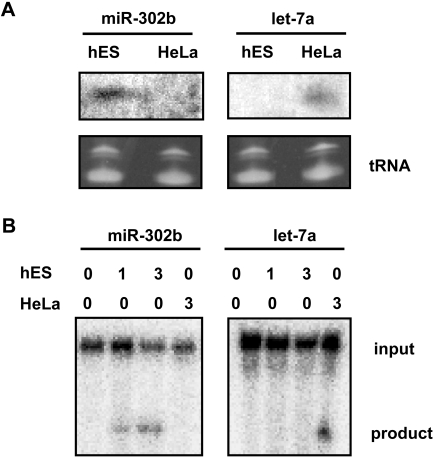
RISC in hES cells retains specific target RNA cleavage activity. (A) Northern blot analysis of miR-302b and miR let-7a expression in hES and HeLa cells, respectively. Total RNA was prepared from hES and HeLa cells and RNAs (10 µg) were loaded onto gels and miRNA bands were resolved by 14% denaturing gels. miRNAs were hybridized with various ^32^P labeled specific DNA probes as indicated. Specific bands were visualized by phosphoImager. tRNA band shows the loading control. (B) Specific target RNA cleavage by RISC programmed with miR-302 and let-7a. RISC activity of miR-302b, a representative miRNA expressed in hES cells and not in HeLa cells, and let-7a was analyzed by incubating cell extracts with a ^32^P-cap-labeled substrate mRNA that was perfectly complementary to miR-302b or let-7a. Cleavage reactions and product analysis were performed as previously described [27]. Labels indicate amount of extracts (0, 1, and 3 µg) from hES and HeLa cells, target RNA and cleavage products.

To determine whether miRNAs in human ES cells assemble into Ago2-containing effector RISCs, we employed the target RNA-cleavage capabilities of RISC when the target and miRNA have perfectly matched complementary sequences. For these experiments, we chose miR-302b because it was abundant in hES cells, thus providing a sensitive assay for RISC activity programmed with miRNAs. Since let-7a is abundant in HeLa, we selected let-7 as a control in our experiments. Fig 4A demonstrates the specific expression of miR-302b and let-7 in hES and HeLa cells, respectively. RISC-mediated target RNA cleavage activity was determined by *in vitro* cleavage of a ^32^P-target mRNA that perfectly matched the miR-302b or let-7 sequence. Our results (Fig 4B) show that ES cell extracts (1 and 3 µg) contained active miR-302b RISC and cleaved the perfectly matched target mRNA, whereas no target cleavage activity was seen in extracts of HeLa cells, which do not express miR-302b. Conversely, let-7 RISC in HeLa extracts cleaved let-7 target RNA while hES RISC did not show detectable activity in this experiment. Taken together, these results demonstrate that miRNAs in human ES cells assemble into a *bona fide* RISC that contains Ago2 and is capable of cleaving target mRNA.

In summary, we have identified hES cell-specific miRNAs and have determined that the evolution of these miRNAs is a recent primate-specific phenomenon, dating back to ∼5 million years. In addition, our results demonstrate that human ES cell differentiation is accompanied by changes in the expression of a unique set of miRNAs, providing a glimpse of a new posttranscriptional regulatory circuitry that may control early development in humans. An exciting direction for future studies would be to determine how these miRNAs modulate expression of target genes during stem cell self-renewal and differentiation.

## Materials and Methods

### Human embryonic stem (hES) cell culture

hES cell line H1 (obtained from WiCell) was used from passages 42 to 50. Details of the hES cell culture protocol and reagents are available at http://www.wicell.org. Briefly, H1 cells were grown in DMEM/F12 medium (Invitrogen; Cat# 11330-032) containing 20% knockout serum replacer (Invitrogen; Cat# 10828), 4 ng/ml of human recombinant basic fibroblast growth factor (bFGF; Invitrogen; Cat# 13256-029), 1 mM L-glutamine (Invitrogen; Cat# 25030081), and 1% non-essential amino acids (Invitrogen; Cat# 11140-050). hES cells were grown on a feeder layer of mouse embryonic fibroblasts (MEF) at passages 1 to 4. MEF were irradiated and seeded at 1.88×10^5^ cells per well in a 6-well plate. After passage 4, hES cells were grown in feeder-free cultures, i.e., on matrigel-coated plates with MEF-conditioned medium plus bFGF as described previously [23]. hES cell culture medium (as described above) was conditioned for 24 h on MEF at 2.12×10^5^ cells/ml and bFGF was added.

### Karyotyping of hES

The karyotypic stability of hES cells was assessed by cytogenetic analysis at various passages. hES cells were treated overnight with 0.005 µg/ml of Karyo Max Colcemid solution in conditioned medium with bFGF (Gibco; Cat# 15212-012, 10 µg/ml). The medium containing colcemid was removed, and 0.3 ml of 0.05% trypsin (Gibco) was added to each well and incubated for 5 min. Conditioned medium (2.5 ml) was added to cells, which were collected in 15-ml conical tubes. The cell suspension was vigorously pipetted, medium was added to bring the final volume to 5 ml, and karyotype analysis was performed. Results of these analyses on hES at passage 49 are shown in Fig S1.

### Immunostaining for pluripotent markers

hES cells were characterized for pluripotency by staining for pluripotency markers as described [24]. Briefly, hES cells were rinsed with 1 ml PBS, fixed with 4% paraformaldehyde in PBS for 30 min at room temperature (RT), and washed with 1 ml PBS. Cells were permeabilized for 5 min at RT by treating with 0.1% Triton X-100 in PBS. Permeabilized cells were treated with 5% goat serum in PBS for 30 min at RT and incubated for 1 h at RT with antibodies to OCT-4 (Santa Cruz) and SSEA-4 (Santa Cruz) in 1.5% goat serum in PBS according to conditions provided with the antibodies. After washing (3×) with 1 ml of PBS, cells were treated with secondary antibodies (Alexa Fluor 488 and 546) diluted 1:100 in 1.5% goat serum in PBS for 1 h at RT. Cell nuclei were stained with Vectashield mounting medium containing DAPI (4′,6 diamidino-2-phenylindole; Vector Laboratories; Cat# H-1200) in PBS. Images were recorded by fluorescence microscopy at 10× magnification. A typical image from these studies is shown in Fig S2.

### Formation of embryoid bodies

Embryoid body formation and characterization were performed as previously described [25], [26]. Briefly, the medium was removed from hES cells, which were washed once with DPBS (2 ml, Gibco). Cell layers were prepared for passage by adding 1 ml of 1 mg/ml Dispase (Gibco) to each well of the 6-well plate, incubating for 5 min at 37°C or 5 min, removing the Dispase, and washing the cells with 2 ml of DPBS. To the washed cells was added 1 ml of complete medium (80% DMEM/F12, 20% knockout serum replacer, 1% non-essential amino acids, 25 µM L-glutamine with B-ME, without bFGF). Cells (including all clumps) were removed with a glass pipette, collected in 15-ml tubes, and centrifuged for 5 min at 1000 rpm at RT. The medium was removed and the pellet was gently resuspended into small clumps. Cells were split and medium was changed every 2 days. The formation and morphology of EBs were analyzed over 14 days by microscopy and immunostaining. The morphology of hES cells and EBs is shown at various times in Fig S3.

### miRNA analysis

Total RNA (20 µg) was isolated from hES cells and EBs and the expression profile for all known miRNAs was analyzed using custom on-chip parallel synthesis microarrays (LC Sciences, TX USA). Each chip contained 7 redundant regions of each miRNA. Each region further contained a miRNA probe region detecting miRNA transcripts listed in the Sanger miRNA database (http://www.sanger.ac.uk/software/Rfam/mirna/). Multiple and T_M_-normalized control probes were included on each chip. Among the control probes, PUC2PM-20B and PUC2MM-20B were the perfectly matched and single-base matched detection probes, respectively. A 20-mer RNA positive control sequence was spiked into the RNA samples before labeling. Hybridization used 100 µL 6xSSPE buffer (0.90 M NaCl, 60 mM Na2HPO4, 6 mM EDTA, pH 6.8) containing 25% formamide at 34 °C. Hybridization was detected by fluorescence labeling with tag-specific Cy3 and Cy5 dyes. Hybridization images were collected using a laser scanner (GenePix 4000B, Molecular Device) and digitized using Array-Pro image analysis software (Media Cybernetics). Data were analyzed by first subtracting the background and then normalizing the signals using a LOWESS filter (locally-weighted regression). Detectable signals with average intensity 3 times higher than background standard deviation and CV (standard deviation/average intensity) less than 0.5 were included in further analysis. Cy3 and Cy5 images were quantified to obtain differential expressions between the hES and EB miRNAs. The images were displayed in pseudo colors to expand the dynamic visual range. For the Cy3 and Cy5 images, as the signal intensity increased from 1 to 65,535, the corresponding color changed from blue to green to yellow to red. The two signals were compared by calculating their ratio (log2 transformed, balanced) and by t-test to determine if they were significantly different. Signals were considered differentially detected if p<0.01. The data represent an average of 3 experiments.

## Supporting Information

Figure S1 hES cells show normal karyotype. A representative image from karyotypic analysis of hES cells shows a normal male karyotype. Examination of 20 metaphase cells showed all normal male 46, XY, with no evidence of structural or numerical abnormalities.
(5.55 MB TIF)

Figure S2 hES cells are pluripotent. hES cells were immunostained for pluripotent markers, OCT4 and SSEA4 [24].
(8.81 MB TIF)

Figure S3 hES cells and EBs show normal morphology over 14 days. The morphology of hES cells and EBs was recorded at days 2, 4, 6, 8, 10, 12, and 14.
(9.51 MB TIF)

Table S1 Name, sequence, and chromosome loci of 276 miRNAs expressed in hES cells. Each miRNA gene sequence was aligned with the human genomic database at Ensemble genome browser (http://www.ensembl.org/Multi/blastview\ version 41, 2006). Matched chromosomal regions with E values less than 0.01 (except marked sequences) are shown. Red indicates a chromosome with a perfectly matched region. Plum indicates matched sequences obtained from the UCSC Genome Browser (http://genome.ucsc.edu). Blue indicates matched sequences calculated from the Sanger miRBase::Sequences (http://microrna.sanger.ac.uk) based on the stem-loop structures.
(0.28 MB PDF)

Table S2 Conservation of 276 hES cell-specific miRNAs in vertebrates. Human miRNA genes were compared with genes of various vertebrate species by multiple alignments to determine the conservation of 276 miRNAs across species. All alignments were tracked from the UCSC Genome Browser (http://genome.ucsc.edu) with the BLAST-Like Alignment Tool (BLAT). The evolutionary conservation of miRNAs was measured in 17 vertebrates, including mammalian, amphibian, bird, and fish species. Multiple alignment of the following gene assemblies were used to generate the analysis track: human (Mar. 2006, hg18), chimpanzee (Nov 2003, panTro1), •macaque (Jan 2006, rheMac2),•mouse (Feb 2006, mm8),•rat (Nov 2004, rn4),•rabbit (May 2005, oryCun1),•dog (May 2005, canFam2),•cow (Mar 2005, bosTau2),•armadillo (May 2005, dasNov1),•elephant (May 2005, loxAfr1),•tenrec (Jul 2005, echTel1),•opossum (Jan 2006, monDom4),•chicken (Feb 2004, galGal2),•frog (Oct 2004, xenTro1),•zebrafish (May 2005, danRer3),•tetraodon (Feb 2004, tetNig1),•fugu (Aug 2002, fr1). Conservation of miRNA genes in 4 species was defined as perfect alignment of at least 3 species among mouse, rat, rabbit, dog and cow (rarely, armadillo, elephant, tenrec or opossum) with a human miRNA sequence. If an miRNA had more than one matched region (repeats) in human chromosomes and only one region was perfectly aligned with other species, it was defined as conserved. Sequence alignments with 2 mismatches or a single mismatch in the miRNA seed region (2–8 nucleotides) were designated as nonconserved.
(0.33 MB PDF)

Table S3 Differential expression levels and evolutionary conservation of hES cell-specific miRNAs during embryoid body (EB) formation. miRNA expression was analyzed in hES cells and EBs after 14 days of differentiation. miRNA expression is arranged by log2 ratio in hES cells/EBs (EB/ES) for 30 of ∼276 miRNAs whose log2 ratio >1. miRNA conservation in various species was determined as described in Table 1.
(0.18 MB PDF)
