# Supplementary figures and images for: Evolutionary Emergence of microRNAs in Human Embryonic Stem Cells
Source: PLoS One. 2008 Jul 30;3(7):e2820. doi: 10.1371/journal.pone.0002820 (PMC2474702; doi:10.1371/journal.pone.0002820)

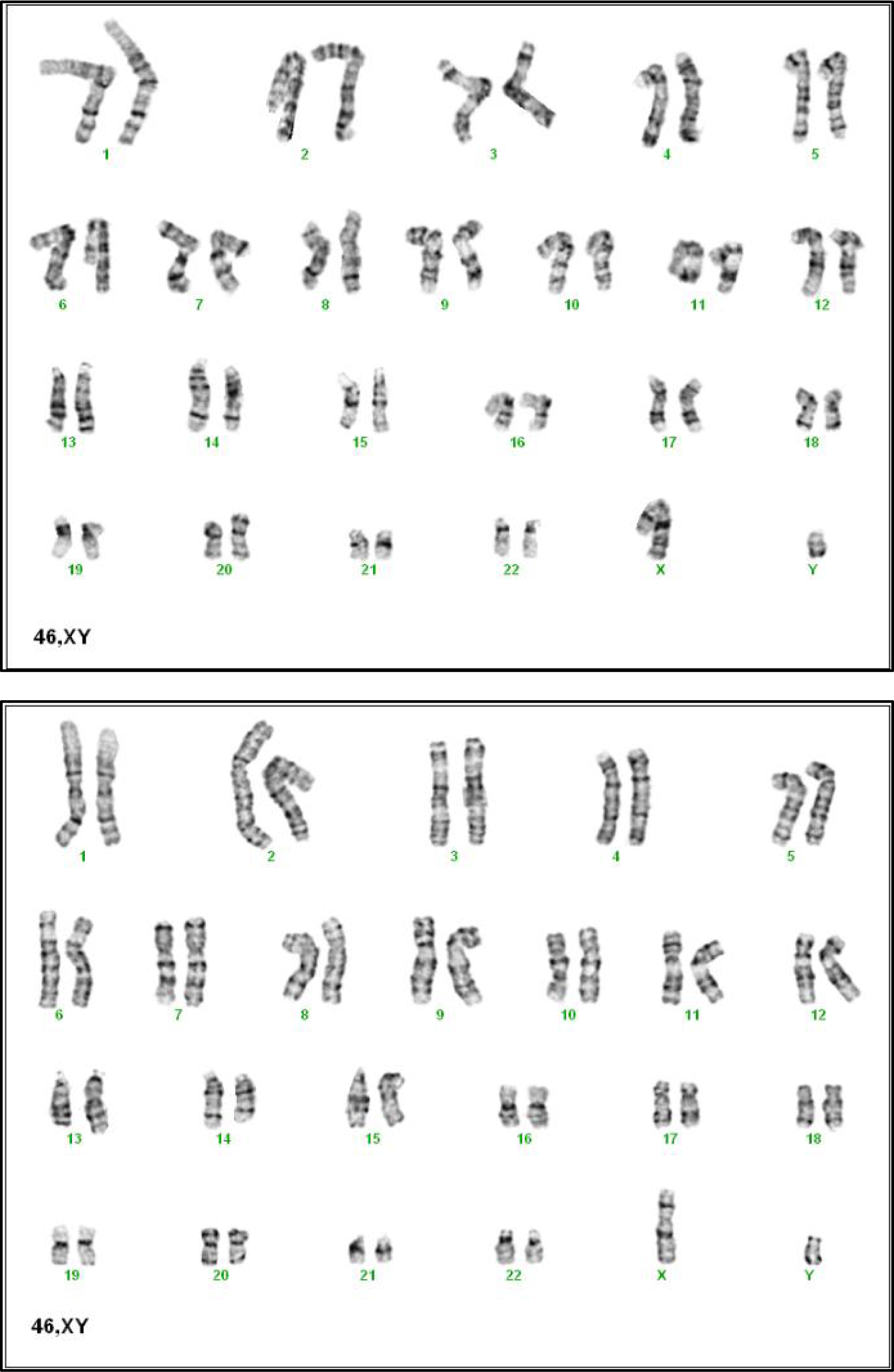

Supplement: Figure S1 — hES cells show normal karyotype. A representative image from karyotypic analysis of hES cells shows a normal male karyotype. Examination of 20 metaphase cells showed all normal male 46, XY, with no evidence of structural or numerical abnormalities. (5.55 MB TIF) [file pone.0002820.s001.tif]

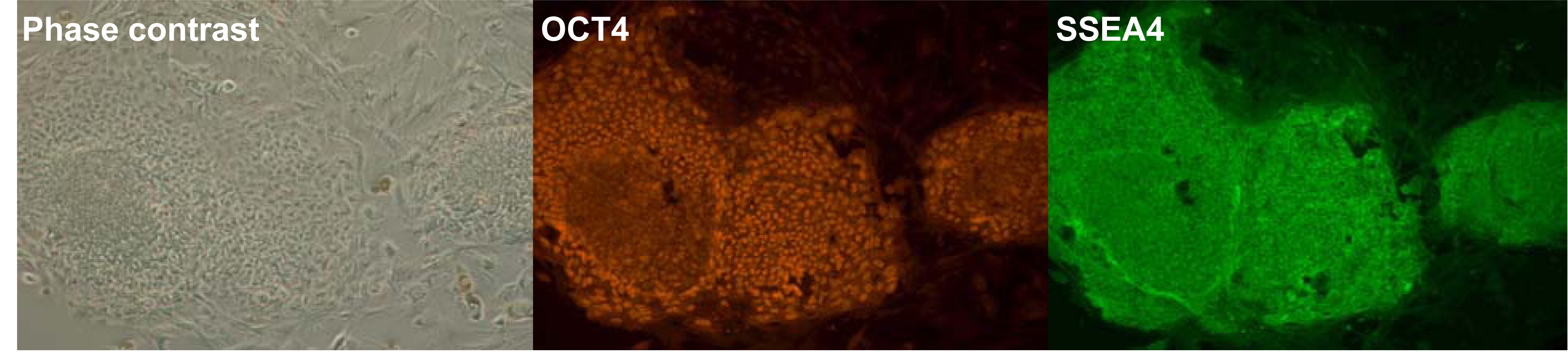

Supplement: Figure S2 — hES cells are pluripotent. hES cells were immunostained for pluripotent markers, OCT4 and SSEA4 [24]. (8.81 MB TIF) [file pone.0002820.s002.tif]

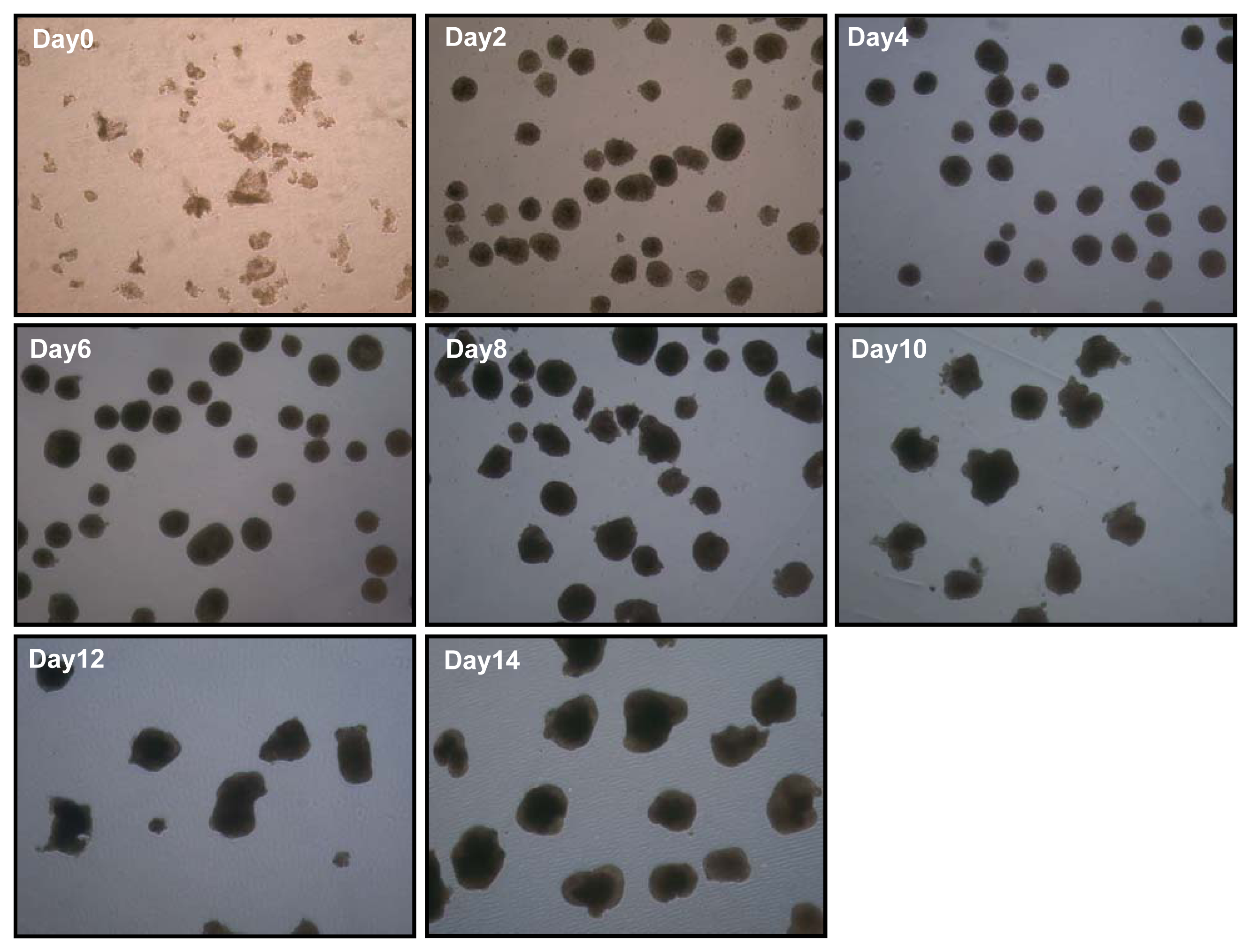

Supplement: Figure S3 — hES cells and EBs show normal morphology over 14 days. The morphology of hES cells and EBs was recorded at days 2, 4, 6, 8, 10, 12, and 14. (9.51 MB TIF) [file pone.0002820.s003.tif]
