# Supplementary material for: Evolutionary Emergence of microRNAs in Human Embryonic Stem Cells
Source: PLoS One. 2008 Jul 30;3(7):e2820. doi: 10.1371/journal.pone.0002820 (PMC2474702; doi:10.1371/journal.pone.0002820)
Supplement: Table S1 — Name, sequence, and chromosome loci of 276 miRNAs expressed in hES cells. Each miRNA gene sequence was aligned with the human genomic database at Ensemble genome browser (http://www.ensembl.org/Multi/blastview\ version 41, 2006). Matched chromosomal regions with E values less than 0.01 (except marked sequences) are shown. Red indicates a chromosome with a perfectly matched region. Plum indicates matched sequences obtained from the UCSC Genome Browser (http://genome.ucsc.edu). Blue indicates matched sequences calculated from the Sanger miRBase::Sequences (http://microrna.sanger.ac.uk) based on the stem-loop structures. (0.28 MB PDF) [file pone.0002820.s004.pdf]

Table S1

miRNA	Sequence	Chromosome:loci[strand](* from UCSC,# from Sanger)
hsa-miR-638	AGGGAUCGCGGGCGGGUGGCGGCCU	*19:10690094-10690119[+]
hsa-miR-302b	UAAGUGCUUCCAUGUUUUAGUAG	4:113789094-113789116[-]
hsa-miR-302d	UAAGUGCUUCCAUGUUUGAGUGU	4:113788611-113788633[-]
hsa-miR-302a	UAAGUGCUUCCAUGUUUUGGUGA	4:113788791-113788813[-]
hsa-miR-302a*	UAAACGUGGAUGUACUUGCUUU	4:113788827-113788848[-]
hsa-miR-20a	UAAAGUGCUUAUAGUGCAGGUAG	13:90801327-90801349[+]
hsa-miR-17-5p	CAAAGUGCUUACAGUGCAGGUAGU	13:90800873-90800896[+],X:133131940-133131961[-]
hsa-miR-302c	UAAGUGCUUCCAUGUUUCAGUGG	4:113788971-113788993[+]
hsa-miR-663	AGGCGGGGCGCCGCGGGACCGC	20:26136879-26136900[+],16:33869178-33869197[+]
hsa-miR-20b	CAAAGUGCUCAUAGUGCAGGUAG	X:133131546-133131568[-]
hsa-miR-106a	AAAAGUGCUUACAGUGCAGGUAGC	X:133131939-133131962[-],13:90800874-90800895[+]
hsa-miR-25	CAUUGCACUUGUCUCGGUCUGA	7:99529130-99529151[-]
hsa-miR-92	UAUUGCACUUGUCCCGGCCUG	13:90801616-90801636[+],X:133131241-133131261[-]
hsa-miR-21	UAGCUUAUCAGACUGAUGUUGA	17:55273416-55273437[+]
hsa-miR-92b	UAUUGCACUCGUCCCGGCCUC	1:153431652-153431672[+]
hsa-miR-26a	UUCAAGUAAUCCAGGAUAGGC	12:56504709-56504729[-],3:37985908-37985928[+]
hsa-miR-15b	UAGCAGCACAUCAUGGUUUACA	3:161605089-161605110[+]
hsa-miR-513	UUCACAGGGAGGUGUCAUUUAU	X:146115106-146115127[-],X:146102744-146102765[-]
hsa-miR-19b	UGUGCAAAUCCAUGCAAAACUGA	13:90801500-90801522[+],X:133131379-33131401[-]
hsa-miR-130a	CAGUGCAAUGUUAAAAGGGCAU	11:57165301-57165322[+]
hsa-miR-200c	UAAUACUGCCGGGUAAUGAUGG	12:6943166-6943187[+]
hsa-miR-93	AAAGUGCUGUUCGUGCAGGUAG	7:99529374-99529395[-]
hsa-miR-612	GCUGGGCAGGGCUUCUGAGCUCCUU	11:64968520-64968544[+],1:11628980-11629002[+],11:194222463-19422482[-]
hsa-miR-16	UAGCAGCACGUAAAUAUUGGCG	3:161605236-161605257[+],13:49521164-49521185[-]
hsa-miR-103	AGCAGCAUUGUACAGGGCUAUGA	20:3846188-3846210[+],5:167920487-167920509[-],10:91342495-91342515[-]
hsa-miR-107	AGCAGCAUUGUACAGGGCUAUCA	10:91342493-91342515[-],20:3846188-3846210[+],5:167920489-167920509[-]
hsa-miR-221	AGCUACAUUGUCUGCUGGGUUUC	X:45490552-45490574[-]
hsa-miR-363	AAUUGCACGGUAUCCAUCUGUA	X:133131078-133131099[-]
hsa-miR-575	GAGCCAGUUGGACAGGAGC	#4:83893529-83893547[-]
hsa-miR-373	GAAGUGCUUCGAUUUUGGGGUGU	19:58983813-58983836[+]
hsa-miR-342	UCUCACACAGAAAUCGCACCCGUC	14:99645805-99645828[+]
hsa-miR-191	CAACGGAAUCCCAAAAGCAGCU	*3:49033109-49033131[-]
hsa-miR-302c*	UUUAACAUGGGGGUACCUGCUG	4:113789007-113789028[-]
hsa-miR-130b	CAGUGCAAUGAUGAAAGGGCAU	22:20337643-20337664[+]
hsa-miR-494	UGAAACAUACACGGGAAACCUCUU	14:100565771-100565794[+]
hsa-miR-106b	UAAAGUGCUGACAGUGCAGAU	7:99529602-99529622[-]
hsa-miR-320	AAAAGCUGGGUUGAGAGGGCGAA	*8:22158431-22158454[-],1:222511372-222511395[-],1:117015931-117015954[+]
hsa-miR-30c	UGUAAACAUCCUACACUCUCAGC	1:40995559-40995581[+],6:72143427-72143449[-]
hsa-miR-594	CCCAUCUGGGGUGGCCUGUGACUUU	7:138676046-138676070[+]
hsa-miR-200b	UAAUACUGCCUGGUAAUGAUGAC	1:1092403-1092425[+]
hsa-miR-26b	UUCAAGUAAUUCAGGAUAGGUU	2:218975624-218975645[+]
hsa-miR-182	UUUGGCAAUGGUAGAACUCACA	7:129197525-129197546[-]
hsa-miR-374	UUAUAAUACAACCUGAUAAGUG	X:73423885-73423906[-]
hsa-miR-30b	UGUAAACAUCCUACACUCAGCU	8:135881995-135882016[-]
hsa-miR-222	AGCUACAUCUGGCUACUGGGUCUC	X:45491383-45491406[-]
hsa-miR-148a	UCAGUGCACUACAGAACUUUGU	7:25956067-25956088[-]
hsa-miR-361	UUAUCAGAAUCUCCAGGGGUAC	X:65045342-85045363[-]
hsa-miR-367	AAUUGCACUUUAGCAAUGGUGA	4:113788482-113788503[-],X:137749831-137749850[+]
hsa-miR-18a	UAAGGUGCAUCUAGUGCAGAUA	13:90801011-90801032[+]

hsa-miR-517a	AUCGUGCAUCCCUUUAGAGUGUU	19:58907387-58907409[+],19:58916184-58916206[+]
hsa-miR-195	UAGCAGCACAGAAAUAUUGGC	17:6861710-6861730[-]
hsa-miR-637	ACUGGGGGCUUUCGGGCUCUGCGU	19:3912427-3912450[-]
hsa-miR-371	GUGCCGCCAUCUUUUGAGUGU	19:58982784-58982804[+]
hsa-miR-183	UAUGGCACUGGUAGAAUUCACUG	7:129202042-129202064[-]
hsa-miR-421	AUCAACAGACAUUAAUUGGGCGC	X:73354952-73354974[-]
hsa-miR-517b	UCGUGCAUCCCUUUAGAGUGUU	19:58907388-58907409[+],19:58916185-58916206[+]
hsa-miR-23b	AUCACAUUGCCAGGGAUUACC	9:96887368-96887388[+]
hsa-miR-498	UUUCAAGCCAGGGGGCGUUUUUC	19:58869296-58869318[+]
hsa-miR-526a	CUCUAGAGGGAAGCACUUUCU	19:58895096-58895116[+],19:58901332-58901352[+],19:58902534-58902554[+], 19:58921994-58922014[+],19:58929958-58929978[+]
hsa-miR-23a	AUCACAUUGCCAGGGAUUUCC	19:13808409-13808429[-]
hsa-miR-372	AAAGUGCUGCGACAUUUGAGCGU	19:58982997-58983019[+]
hsa-miR-602	GACACGGGCGACAGCUGCGGCCC	*9:139852707-139852729[+]
hsa-miR-18b	UAAGGUGCAUCUAGUGCAGUUA	X:133131781-133131802[-]
hsa-miR-517*	CCUCUAGAUGGAAGCACUGUCU	19:58907348-58907369[+],19:58916147-58916168[+],19:58936398-58936419[+]
hsa-miR-302b*	ACUUUAACAUGGAAGUGCUUUCU	4:113789130-113789152[-]
hsa-miR-335	UCAAGAGCAAUAACGAAAAAUGU	7:129923203-129923225[+]
hsa-miR-129	CUUUUUGCGGUCUGGGCUUGC	7:127635165-127635185[+],11:43559534-43559554[+]
hsa-miR-19a	UGUGCAAAUCUAUGCAAAACUGA	13:90801194-90801216[+]
hsa-miR-518c*	CAAAGCGCUUCUCUUUAGAGUG	19:58903862-58903883[+]
hsa-miR-524*	CUACAAAGGGAAGCACUUUCUC	19:58906083-58906104[+]
hsa-miR-516-5p	CAUCUGGAGGUAAGAAGCACUUU	19:58920522-58920544[+],19:58931925-58931947[+]
hsa-miR-185	UGGAGAGAAAGGCAGUUC	22:18400676-18400693[+] E=0.098
hsa-miR-658	GGCGGAGGGAAGUAGGUCCGUUGGU	22:36570240-36570264[-]
hsa-miR-425-5p	AAUGACACGAUCACUCCCGUUGA	3:49032636-49032658[-]
hsa-miR-125a	UCCCUGAGACCCUUUAACCUGUG	19:56888333-56888355[+]
hsa-miR-128b	UCACAGUGAACCGGUCUCUUUC	3:35761023-35761044[+],2:136139486-136139506[+]
hsa-miR-128a	UCACAGUGAACCGGUCUCUUUU	2:136139486-136139507[+],3:35761023-35761043[+]
hsa-miR-29a	UAGCACCAUCUGAAAUCGGUU	7:130212048-130212068[-]
hsa-miR-198	GGUCCAGAGGGGAGAUAGG	3:1215597243-121597261[-]
hsa-miR-422b	CUGGACUUGGAGUCAGAAGGCC	5:149092624-149092645[+},3:32002802-32002823[-],11:120022935-120022956[-]
hsa-miR-151	ACUAGACUGAAGCUCCUUGAGG	8:141811868-141811889[-],7:150838585-150838606[-],17:17715008-17715028[+]
hsa-miR-148b	UCAGUGCAUCACAGAACUUUGU	12:53017329-53017350[+]
hsa-miR-28	AAGGAGCUCACAGUCUAUUGAG	3:189889276-189889297[+],8:131558554-131558573[+],8:119421382-119421401[-]
hsa-miR-518f*	CUCUAGAGGGAAGCACUUUCUCU	19:58895096-58895118[+],19:58901332-58901352[+],19:58902534-58902554[+], 19:58921994-58922014[+],19:58929958-58929978[+]
hsa-miR-30d	UGUAAACAUCCCCGACUGGAAG	8:135886344-135886365[-]
hsa-miR-126	UCGUACCGUGAGUAAUAAUGC	9:138684926-138684946[+]
hsa-miR-27b	UUCACAGUGGCUAAGUUCUGC	9:96887608-96887628[+]
hsa-miR-24	UGGCUCAGUUCAGCAGGAACAG	9:96888167-96888188[+],19:13808103-13808124[-]
hsa-miR-187	UCGUGUCUUGUGUUGCAGCCG	18:31738797-31738817[-]
hsa-miR-512-3p	AAGUGCUGUCAUAGCUGAGGUC	19:58861795-58861816[+],19:58864279-58864300[+]
hsa-miR-492	AGGACCUGCGGGACAAGAUUCUU	12:93752334-93752356[+],12:9171765-9171785[+]
hsa-miR-526b	CUCUUGAGGGAAGCACUUUCUGUU	19:58889472-58889495[+],19:58901332-58901355[+],19:58902534-58902557[+], 19:58903825-58903848[+],19:58921994-58922017[+],19:58929958-58929981[+]
hsa-miR-650	AGGAGGCAGCGCUCUCAGGAC	*22:21495284-21495305[+],22:21431199-21431220[+]
hsa-miR-101	UACAGUACUGUGAUAACUGAAG	1:65296712-65296733[-],9:4840345-4840366[+]
hsa-miR-564	AGGCACGGUGUCAGCAGGC	#3:44878399-44878417[+]
hsa-miR-574	CACGCUCAUGCACACACCCAC	*4:38546108-38546128[+]
hsa-miR-520h	ACAAAGUGCUUCCCUUUAGAGU	19:58917286-58917307[+],19:58937632-58937653[+]
hsa-miR-520g	ACAAAGUGCUUCCCUUUAGAGUGU	19:58917286-58917309[+],19:58937632-58937653[+]

hsa-miR-30a-5p	UGUAAACAUCCUCGACUGGAAG	6:72170019-72170040[-]
hsa-miR-193b	AACUGGCCCUCAAAGUCCCGCUUU	16:14305374-14305398[+]
hsa-miR-301	CAGUGCAAUAGUAUUGUCAAAGC	17:54583292-54583314[-]
hsa-miR-565	GGCUGGCUCGCGAUGUCUGUUU	3:45705483-45705504[-]
hsa-miR-7	UGGAAGACUAGUGAUUUUGUUG	19:4721712-4721733[+],15:86956091-86956112[+],9:85774548-85774569[-]
hsa-miR-135a	UAUGGCUUUUUAUUCCUAUGUGA	3:52303326-52303348[-],12:96481743-96481765[+]
hsa-miR-422a	CUGGACUUAGGGUCAGAAGGCC	15:61950240-61950261[-]
hsa-miR-99b	CACCCGUAGAACCGACCUUGCG	*19:56887683-56887704[+]
hsa-miR-206	UGGAAUGUAAGGAAGUGUGUGG	6:52117158-52117179[+]
hsa-miR-365	UAAUGCCCCUAAAAAUCCUUAU	16:14310698-14310719[+],17:26926610-26926631[+]
hsa-miR-572	GUCCGCUCGGCGGUGGCCCA	4:10979609-10979628[+],16:2257803-2257822[-]
hsa-miR-505	GUCAACACUUGCUGGUUUCCUC	X:138833984-138834005[-]
hsa-miR-146b	UGAGAACUGAAUUCCAUAGGCU	10:104186267-104186288[+]
hsa-miR-517c	AUCGUGCAUCCUUUUAGAGUGU	19:58936435-58936456[+]
hsa-miR-124a	UUAAGGCACGCGGUGAAUGCCA	20:61280348-61280369[+],8:65454320-65454341[+],8:9798320-9798341[-]
hsa-miR-27a	UUCACAGUGGCUAAGUUCCGC	19:13808261-13808281[-]
hsa-miR-519d	CAAAGUGCCUCCCUUUAGAGUGU	19:58908466-58908488[+]
hsa-miR-324-3p	CCACUGCCCCAGGUGCUGCUGG	17:7067351-7067372[-]
hsa-miR-210	CUGUGCGUGUGACAGCGGCUGA	11:558112-558133[-]
hsa-miR-373*	ACUCAAAAUGGGGGCGCUUUCC	19:58983776-58983797[+]
hsa-miR-497	CAGCAGCACACUGUGGUUUGU	17:6862022-6862042[-]
hsa-miR-636	UGUGCUUGCUCGUCCCGCCCGCAG	17:72244142-72244165[-]
hsa-miR-484	UCAGGCUCAGUCCCCUCCCGAU	16:15644659-15644680[+]
hsa-miR-205	UCCUUCAUUCCACCGGAGUCUG	1:207672134-207672155[+]
hsa-miR-296	AGGGCCCCCCCUCAAUCCUGU	20:56826111-56826131[-]
hsa-miR-186	CAAAGAAUUCUCCUUUUGGGCUU	1:71305951-71305973[-]
hsa-miR-519c	AAAGUGCAUCUUUUUAGAGGAU	19:58881588-58881609[+]
hsa-miR-30e-3p	CUUUCAGUCGGAUGUUUACAGC	1:40992672-40992693[+]
hsa-miR-15a	UAGCAGCACAUAAUGGUUUGUG	13:49521304-49521325[-],7:3516341-3516360[-]
hsa-miR-214	ACAGCAGGCACAGACAGGCAG	1:170374580-170374600[-]
hsa-miR-192	CUGACCUAUGAAUUGACAGCC	11:64415251-64415271[-]
hsa-miR-331	GCCCCUGGGCCUAUCCUAGAA	12:94226387-94226407[+]
hsa-miR-223	UGUCAGUUUGUCAAAUACCCC	X:65155504-65155524[+]
hsa-miR-34a	UGGCAGUGUCUUAGCUGGUUGU	1:9134380-9134402[-]
hsa-miR-197	UUCACCACCUUCUCCACCCAGC	1:109943085-109943106[+]
hsa-miR-520d	AAAGUGCUUCUCUUUGGUGGGUU	19:58915215-58915237[+]
hsa-miR-601	UGGUCUAGGAUUGUUGGAGGAG	9:125204667-125204688[-]
hsa-miR-598	UACGUCAUCGUUGUCAUCGUCA	8:10930141-10930162[-]
hsa-miR-191*	GCUGCGCUUGGAUUUCGUCCCC	3:49033068-49033089[-]
hsa-miR-515-5p	UUCUCCAAAAGAAAGCACUUUCUG	19:58874082-58874105[+],19:58880088-58880111[+]
hsa-miR-557	GUUUGCACGGGUGGGCCUUGUCU	1:166611446-166611468[+]
hsa-miR-520c	AAAGUGCUUCCUUUUAGAGGGUU	19:58896333-58896355[+],19:58902572-58902594[+],19:58877279-58877300[+], 19:58889510-5888952[+]
hsa-miR-149	UCUGGCUCCGUGUCUUCACUCC	2:241044105-241044126[+]
hsa-miR-489	AGUGACAUCACAUAUACGGCAGC	7:92951195-92951217[-]
hsa-miR-31	GGCAAGAUGCUGGCAUAGCUG	9:21502156-21502176[-]
hsa-miR-525	CUCCAGAGGGAUGCACUUUCU	19:58892613-58892633[+]
hsa-miR-18a*	ACUGCCCUAAGUGCUCCUUCU	13:90801052-90801072[+]
hsa-miR-519e*	AAAGUGCCUCCUUUUAGAGUGU	19:58875056-58875077[+]
hsa-miR-520f	AAGUGCUUCCUUUUAGAGGGUU	19:58877279-58877300[+],19:58896334-58896355[+],19:58902573-58902594[+]
hsa-miR-593	AGGCACCAGCCAGGCAUUGCUCAGC	*7:127509163-127509188[+]
hsa-miR-125b	UCCCUGAGACCCUAACUUGUGA	21:16884444-16884465[+],11:121475727-121475748[-]

hsa-miR-204	UUCCCUUUGUCAUCCUAUGCCU	9:72614767-72614788[-]
hsa-miR-623	AUCCCUUGCAGGGGCUGUUGGGU	13:98806401-98806423[+]
hsa-miR-519b	AAAGUGCAUCCUUUUAGAGGUUU	19:58890329-58890351[+]
hsa-miR-628	UCUAGUAAGAGUGGCAGUCG	15:53452445-53452464[-]
hsa-miR-22	AAGCUGCCAGUUGAAGAACUGU	17:1563958-1563979[-]
hsa-miR-194	UGUAACAGCAACUCCAUGUGGA	11:64415452-64415473[-],1:218358171-218358192[-]
hsa-miR-199a*	UACAGUAGUCUGCACAUUGGUU	19:10789106-10789121[-],1:170380318-170380339[-],9:130046846-130046867[-]
hsa-miR-340	UCCGUCUCAGUUACUUUAUAGCC	5:179374924-179374946[-]
hsa-miR-520a*	AAAGUGCUUCCCUUUGGACUGU	19:58885999-58886020[+}
hsa-miR-615	UCCGAGCCUGGGUCUCCCUCU	12:52714061-52714081[+]
hsa-miR-200a	UAACACUGUCUGGUAACGAUGU	1:1093159-1093180[+]
hsa-miR-155	UUAAUGCUAAUCGUGAUAGGGG	21:25868166-25868187[+]
hsa-miR-660	UACCCAUUGCAUAUCGGAGUUG	X:49664604-49664625[+]
hsa-miR-181b	AACAUUCAUUGCUGUCGGUGGG	9:126495825-126495846[+],1:197094678-197094699[-]
hsa-miR-518b	CAAAGCGCUCCCCUUUAGAGGU	19:58897853-58897874[+]
hsa-miR-532	CAUGCCUUGAGUGUAGGACCGU	X:49654513-49654534[+]
hsa-miR-30e-5p	UGUAAACAUCCUUGACUGGA	1:40992630-40992649[+]
hsa-miR-560	GCGUGCGCCGGCCGGCCGCC	*2:132731985-132732005[-],20:26137832-26137852[-],Y:10643119-10643139[+]
hsa-miR-362	AAUCCUUGGAACCUAGGUGUGAGU	X:49660316-49660339[+]
hsa-miR-425-3p	AUCGGGAAUGUCGUGUCCGCC	3:49032597-49032617[-]
hsa-miR-9*	UAAAGCUAGAUAACCGAAAGU	1:154656770-154656790[-],15:87712307-87712327[+]-,5:87998440-87998460[-]
hsa-miR-625	AGGGGGAAAGUUCUAUAGUCCU	14:65007587-65007608[+],14:65007622-65007643[-]
hsa-miR-518f	AAAGCGCUUCUCUUUAGAGGA	19:58895134-58895154[+]
hsa-miR-429	UAAUACUGUCUGGUAAAACCGU	1:1094298-1094319[+]
hsa-miR-141	UAACACUGUCUGGUAAAGAUGG	12:6943579-6943600[+]
hsa-miR-345	UGCUGACUCCUAGUCCAGGGC	14:99843965-99843985[+]
hsa-miR-424	CAGCAGCAAUUCAUGUUUUGAA	X:133508376-133508397[-]
hsa-miR-150	UCUCCCAACCCUUGUACCAGUG	19:54695901-54695922[-]
hsa-miR-522	AAAAUGGUUCCCUUUAGAGUGUU	19:58946330-58946352[+]
hsa-miR-224	CAAGUCACUAGUGGUUCCGUUUA	X:150877757-150877779[-]
hsa-miR-520b	AAAGUGCUUCCUUUUAGAGGG	19:58896333-58896353[+],19:58902572-58902592[+],19:58877279-58877298[+],<br>19:58889510-58889529[+]
hsa-miR-9	UCUUUGGUUAUCUAGCUGUAUGA	1:154656808-154656830[-],15:87712267-87712289[+]-,5:87998476-87998498[-]
hsa-miR-202	AGAGGUAUAGGGCAUGGGAAAA	*10:134911030-134911052[-]
hsa-miR-188	CAUCCCUUGCAUGGUGGAGGGU	X:49654863-49654884[+]
hsa-miR-200a*	CAUCUUACCGGACAGUGCUGGA	1:1093121-1093142[+]
hsa-miR-518e	AAAGCGCUUCCCUUCAGAGUGU	19:58924957-58924978[+]
hsa-miR-142-5p	CAUAAAGUAGAAAGCACUAC	17:53763644-53763663[-]
hsa-miR-423	AGCUCGGUCUGAGGCCCCUCAG	17:25468275-25468296[+}
hsa-miR-181a	AACAUUCAACGCUGUCGGUGAGU	9:126494580-126494602[+],1:197094860-197094882[-]
hsa-miR-526c	CUCUAGAGGGAAGCGCUUUCUGUU	19:58881550-58881573[+],19:58890291-58890314[+],19:58893466-58893489[+],<br>19:58924919-58924942[+],19:58946292-58946315[+],19:58947477-58947500[+],<br>19:58897820-58897839[+],19:58896295-58896318[+],19:58957425-58957448[+]
hsa-miR-17-3p	ACUGCAGUGAAGGCACUUGU	13:90800910-90800929[+]
hsa-miR-181d	AACAUUCAUUGUUGUCGGUGGGUU	19:13846724-13846747[+]
hsa-miR-563	AGGUUGACAUACGUUUCCC	3:15890333-15890351[+], E=0.025
hsa-miR-629	GUUCUCCCAACGUAAGCCCAGC	15:68158780-68158801[-]
hsa-miR-203	GUGAAAUGUUUAGGACCACUAG	14:103653559-103653580[+]
hsa-miR-589	UCAGAACAAAUGCCGGUUCCCAGA	7:5501991-5502014[+]
hsa-miR-452	UGUUUGCAGAGGAAACUGAGAC	X:150878803-150878824[-]
hsa-miR-654	UGGUGGGCCGCAGAACAUGUGC	14:100576324-100576345[+]
hsa-miR-145	GUCCAGUUUUCCCAGGAAUCCCUU	5:148790417-148790440[+]

hsa-miR-526b*	AAAGUGCUUCCUUUUAGAGGC	19:58889510-58889530[+],19:58896333-58896352[+],19:58902572-58902591[+]
hsa-miR-324-5p	CGCAUCCCCUAGGGCAUUGGUGU	17:7067385-7067407[-]
hsa-miR-146a	UGAGAACUGAAUUCCAUGGGUU	5:159844957-159844978[+]
hsa-miR-652	AAUGGCGCCACUAGGGUUGUGCA	X:109185273-109185295[+]
hsa-miR-515-3p	GAGUGCCUUCUUUUGGAGCGU	19:58874119-58874139[+],19:58880125-58880145[+]
hsa-miR-519a	AAAGUGCAUCCUUUUAGAGUGUUAC	19:58947515-58947539[+],19:58957463-58957487[+],19:58936438-58936459[+], 19:58875056-58875080[+]
hsa-miR-455	UAUGUGCCUUUGGACUACAUCG	9:116011550-116011571[+]
hsa-miR-105	UCAAAUGCUCAGACUCCUGU	X:151313589-151313608[-],X:151311396-151311415[-]
hsa-miR-143	UGAGAUGAAGCACUGUAGCUCA	5:148788734-148788755[+}
hsa-miR-486	UCCUGUACUGAGCUGCCCCGAG	8:41637119-41637140[+],8:41637159-41637180[-]
hsa-miR-542-5p	UCGGGGAUCAUCAUGUCACGAG	X:133503097-133503118[-]
hsa-miR-363*	CGGGUGGAUCACGAUGCAAUUU	X:133131121-133131142[-]
hsa-miR-132	UAACAGUCUACAGCCAUGGUCG	17:1899973-1899994[-]
hsa-miR-152	UCAGUGCAUGACAGAACUUGGG	17:43469538-43469559[-]
hsa-miR-500	AUGCACCUGGGCAAGGAUUCUG	X:49659830-49659851[+],X:49665998-49666017[+]
hsa-miR-122a	UGGAGUGUGACAAUGGUGUUUGU	18:54269300-54269322[+],10:29373153-29373172[+]
hsa-miR-659	CUUGGUUCAGGGAGGGUCCCCA	22:36573646-36573667[-]
hsa-miR-451	AAACCGUUACCAUUACUGAGUUU	17:24212546-24212568[-]
hsa-miR-30a-3p	CUUUCAGUCGGAUGUUUGCAGC	6:72169978-72169999[-]
hsa-miR-590	GAGCUUAUUCAUAAAAGUGCAG	7:73243479-73243500[+],9:11744034-11744053[+]
hsa-miR-346	UGUCUGCCCGCAUGCCUGCCUCU	10:88014484-88014506[-]
hsa-miR-512-5p	CACUCAGCCUUGAGGGCACUUUC	19:58861758-58861780[+],19:58864242-58864264[+]
hsa-miR-641	AAAGACAUAGGAUAGAGUCACCUC	19:45480350-45480373[-]
hsa-miR-330	GCAAAGCACACGGCCUGCAGAGA	*19:50834107-50834129[-],2:31150573-31150594[+]
hsa-miR-502	AUCCUUGCUAUCUGGGUGCUA	X:49665961-49665981[+]
hsa-miR-96	UUUGGCACUAGCACAUUUUUGC	7:129201816-129201837[-]
hsa-miR-634	AACCAGCACCCCAACUUUGGAC	*17:62213712-62213733[+]
hsa-miR-215	AUGACCUAUGAAUUGACAGAC	1:218357881-218357901[-]
hsa-miR-133b	UUGGUCCCCUUCAACCAGCUA	6:52121746-52121766[+],20:60572623-60572642[+],18:17659672-17659691[-]
hsa-miR-501	AAUCCUUUGUCCCUGGGUGAGA	X:49661083-49661104[+]
hsa-miR-550	UGUCUUACUCCCUCAGGCACAU	7:30295995-30296016[+],7:32739178-32739199[+],7:29686889-29686910[-]
hsa-miR-519e	AAAGUGCCUCCUUUUAGAGUGU	19:58875056-58875077[+]
hsa-miR-218	UUGUGCUUGAUCUAACCAUGU	5:168127794-168127814[-],4:20139020-20139040[+]
hsa-miR-212	UAACAGUCUCCAGUCACGGCC	17:1900334-1900354[-]
hsa-miR-133a	UUGGUCCCCUUCAACCAGCUGU	20:60572623-60572644[+],18:17659670-17659691[-],6:52121746-52121765[+]
hsa-miR-503	UAGCAGCGGGAACAGUUCUGCAG	X:133508067-133508089[-]
hsa-miR-378	CUCCUGACUCCAGGUCCUGUGU	5:149092585-149092606[+]
hsa-miR-382	GAAGUUGUUCGUGGUGGAUUCG	14:100590406-100590427[+]
hsa-miR-520e	AAAGUGCUUCCUUUUUGAGGG	19:58870830-58870850[+]
hsa-miR-592	UUGUGUCAAUAUGCGAUGAUGU	7:126485438-126485459[-]
hsa-miR-631	AGACCUGGCCCAGACCUCAGC	*15:73433044-73433064[-]
hsa-miR-527	CUGCAAAGGGAAGCCCUUUCU	19:58926085-58926105[+],19:58934414-58934434[+],19:58949097-58949117[+]
hsa-miR-450	UUUUUGCGAUGUGUUCCUAAUA	X:133502261-133502282[+],X:133502090-133502111[+]
hsa-miR-127	UCGGAUCCGUCUGAGCUUGGCU	14:100419125-100419146[+]
hsa-miR-495	AAACAAACAUGGUGCACUUCUUU	14:100569894-100569916[+]
hsa-miR-100	AACCCGUAGAUCCGAACUUGUG	11:121528193-121528214[-]
hsa-miR-584	UUAUGGUUUGCCUGGGACUGAG	5:148422129-148422150[-],4:40667603-40667622[-]
hsa-miR-409-3p	CGAAUGUUGCUCGGUGAACCCCU	14:100601435-100601457[+]
hsa-miR-504	AGACCCUGGUCUGCACUCUAU	X:137577588-137577608[-]
hsa-miR-647	GUGGCUGCACUCACUUCCUUC	20:62044488-62044508[-],10:50531618-50531637[-]
hsa-miR-328	CUGGCCCUCUCUGCCCUUCCGU	16:65793731-65793752[-]

hsa-miR-29c	UAGCACCAUUUGAAAUCGGU	1:206041835-206041854[-]
hsa-miR-617	AGACUUCCCAUUUGAAGGUGGC	12:79750503-79750524[-]
hsa-miR-370	GCCUGCUGGGGUGGAACCUGG	14:100447276-100447296[+]
hsa-miR-624	UAGUACCAGUACCUUGUGUUCA	14:30553663-30553684[-]
hsa-miR-524	GAAGGCGCUUCCCUUUGGAGU	19:58906120-58906140[+]
hsa-miR-608	AGGGGUGGUGUUGGGACAGCUCCGU	10:102724747-102724771[+]
hsa-miR-520d*	UCUACAAAGGGAAGCCCUUUCUG	19:58915176-58915198[+]
hsa-miR-453	GAGGUUGUCCGUGGUGAGUUCG	14:100592322-100592343[+]
hsa-miR-510	UACUCAGGAGAGUGGCAAUCACA	X:146161587-146161609[-]
hsa-miR-551b	GCGACCCAUACUUGGUUUCAG	3:169752396-169752416[+]
hsa-miR-199a	CCCAGUGUUCAGACUACCUGUUC	19:10789145-10789167[-],1:170380355-170380377[-]
hsa-miR-622	ACAGUCUGCUGAGGUUGGAGC	13:89681497-89681517[+],12:51631811-51631831[+]
hsa-miR-339	UCCCUGUCCUCCAGGAGCUCA	20:559417-559437[-],22:31074124-31074144[-],7:1029154-1029174[-], 22:30854337-30854356[+]
hsa-miR-448	UUGCAUAUGUAGGAUGUCCCAU	X:113964343-113964364[+]
hsa-miR-483	UCACUCCUCUCCUCCCGUCUUCU	11:2111946-2111968[-],8:58828779-58828800[-]
hsa-miR-596	AAGCCUGCCCGGCUCCUCGGG	*8:1752819-1752839[+]
hsa-miR-431	UGUCUUGCAGGCCGUCAUGCA	14:100417116-100417136[+]
hsa-miR-211	UUCCCUUUGUCAUCCUUCGCCU	15:29144590-29144611[-]
hsa-miR-491	AGUGGGGAACCCUUCCAUGAGGA	9:20706119-20706141[+]
hsa-let-7a	UGAGGUAGUAGGUUGUAUAGUU	22:44887296-44887317[+],9:95978065-95978086[+],11:121522486-121522507[-]
hsa-let-7f	UGAGGUAGUAGAUUGUAUAGUU	X:53600932-53600953[-],9:95978456-95978477[+]
hsa-let-7e	UGAGGUAGGAGGUUGUAUAGU	19:56887858-56887878[+]
hsa-let-7b	UGAGGUAGUAGGUUGUGUGGUU	22:44888235-44888256[+],5:142470242-142470261[+]
hsa-let-7i	UGAGGUAGUAGUUUGUGCUGU	12:61283738-61283758[+]
hsa-let-7c	UGAGGUAGUAGGUUGUAUGGUU	21:16834029-16834050[+]
hsa-let-7d	AGAGGUAGUAGGUUGCAUAGU	9:95980944-95980964[+]
hsa-let-7g	UGAGGUAGUAGUUUGUACAGU	3:52277393-52277413[-]
