# Supplementary material for: Evolutionary Emergence of microRNAs in Human Embryonic Stem Cells
Source: PLoS One. 2008 Jul 30;3(7):e2820. doi: 10.1371/journal.pone.0002820 (PMC2474702; doi:10.1371/journal.pone.0002820)
Supplement: Table S2 — Conservation of 276 hES cell-specific miRNAs in vertebrates. Human miRNA genes were compared with genes of various vertebrate species by multiple alignments to determine the conservation of 276 miRNAs across species. All alignments were tracked from the UCSC Genome Browser (http://genome.ucsc.edu) with the BLAST-Like Alignment Tool (BLAT). The evolutionary conservation of miRNAs was measured in 17 vertebrates, including mammalian, amphibian, bird, and fish species. Multiple alignment of the following gene assemblies were used to generate the analysis track: human (Mar. 2006, hg18), chimpanzee (Nov 2003, panTro1), •macaque (Jan 2006, rheMac2),•mouse (Feb 2006, mm8),•rat (Nov 2004, rn4),•rabbit (May 2005, oryCun1),•dog (May 2005, canFam2),•cow (Mar 2005, bosTau2),•armadillo (May 2005, dasNov1),•elephant (May 2005, loxAfr1),•tenrec (Jul 2005, echTel1),•opossum (Jan 2006, monDom4),•chicken (Feb 2004, galGal2),•frog (Oct 2004, xenTro1),•zebrafish (May 2005, danRer3),•tetraodon (Feb 2004, tetNig1),•fugu (Aug 2002, fr1). Conservation of miRNA genes in 4 species was defined as perfect alignment of at least 3 species among mouse, rat, rabbit, dog and cow (rarely, armadillo, elephant, tenrec or opossum) with a human miRNA sequence. If an miRNA had more than one matched region (repeats) in human chromosomes and only one region was perfectly aligned with other species, it was defined as conserved. Sequence alignments with 2 mismatches or a single mismatch in the miRNA seed region (2–8 nucleotides) were designated as nonconserved. (0.33 MB PDF) [file pone.0002820.s005.pdf]

Table S2

miRNA	Sequence	4 species	rhesus	chimpanzee
hsa-miR-638	AGGGAUCGCGGGCGGGUGGCGGCCU	NC	C	NC
hsa-miR-302b	UAAGUGCUUCCAUGUUUUAGUAG	C	C	C
hsa-miR-302d	UAAGUGCUUCCAUGUUUGAGUGU	C	C	C
hsa-miR-302a	UAAGUGCUUCCAUGUUUUGGUGA	NC	C	C
hsa-miR-302a*	UAAACGUGGAUGUACUUGCUUU	C	C	C
hsa-miR-20a	UAAAGUGCUUAUAGUGCAGGUAG	C	NC	C
hsa-miR-17-5p	CAAAGUGCUUACAGUGCAGGUAGU	C	C	C
hsa-miR-302c	UAAGUGCUUCCAUGUUUCAGUGG	C	C	C
hsa-miR-663	AGGCGGGGCGCCGCGGGACCGC	NC	NC	C
hsa-miR-20b	CAAAGUGCUCAUAGUGCAGGUAG	C	C	C
hsa-miR-106a	AAAAGUGCUUACAGUGCAGGUAGC	C	C	C
hsa-miR-25	CAUUGCACUUGUCUCGGUCUGA	C	C	NC
hsa-miR-92	UAUUGCACUUGUCCCGGCCUG	C	C	C
hsa-miR-21	UAGCUUAUCAGACUGAUGUUGA	C	C	C
hsa-miR-92b	UAUUGCACUCGUCCCGGCCUC	C	C	NC
hsa-miR-26a	UUCAAGUAAUCCAGGAUAGGC	C	C	C
hsa-miR-15b	UAGCAGCACAUCAUGGUUUACA	C	C	C
hsa-miR-513	UUCACAGGGAGGUGUCAUUUAU	NC	C	C
hsa-miR-19b	UGUGCAAAUCCAUGCAAAACUGA	C	C	C
hsa-miR-130a	CAGUGCAAUGUUAAAAGGGCAU	C	C	C
hsa-miR-200c	UAAUACUGCCGGGUAAUGAUGG	C	C	NC
hsa-miR-93	AAAGUGCUGUUCGUGCAGGUAG	C	C	C
hsa-miR-612	GCUGGGCAGGGCUUCUGAGCUCCUU	NC	NC	C
hsa-miR-16	UAGCAGCACGUAAAUAUUGGCG	C	C	C
hsa-miR-103	AGCAGCAUUGUACAGGGCUAUGA	C	C	C
hsa-miR-107	AGCAGCAUUGUACAGGGCUAUCA	C	C	C
hsa-miR-221	AGCUACAUUGUCUGCUGGGUUUC	C	C	C
hsa-miR-363	AAUUGCACGGUAUCCAUCUGUA	C	C	NC
hsa-miR-575	GAGCCAGUUGGACAGGAGC	NC	NC	C
hsa-miR-373	GAAGUGCUUCGAUUUUGGGGUGU	NC	C	C
hsa-miR-342	UCUCACACAGAAAUCGCACCCGUC	C	C	C
hsa-miR-191	CAACGGAAUCCCAAAAGCAGCU	C	C	C
hsa-miR-302c*	UUUAACAUGGGGGUACCUGCUG	C	C	C
hsa-miR-130b	CAGUGCAAUGAUGAAAGGGCAU	C	C	C
hsa-miR-494	UGAAACAUACACGGGAAACCUCUU	C	C	C
hsa-miR-106b	UAAAGUGCUGACAGUGCAGAU	C	C	C
hsa-miR-320	AAAAGCUGGGUUGAGAGGGCGAA	C	C	C
hsa-miR-30c	UGUAAACAUCCUACACUCUCAGC	C	C	C
hsa-miR-594	CCCAUCUGGGGUGGCCUGUGACUUU	NC	NC	C
hsa-miR-200b	UAAUACUGCCUGGUAAUGAUGAC	C	NC	NC
hsa-miR-26b	UUCAAGUAAUUCAGGAUAGGUU	C	C	C
hsa-miR-182	UUUGGCAAUGGUAGAACUCACA	C	C	C
hsa-miR-374	UUAUAAUACAACCUGAUAAGUG	C	C	C
hsa-miR-30b	UGUAAACAUCCUACACUCAGCU	C	C	C
hsa-miR-222	AGCUACAUCUGGCUACUGGGUCUC	C	C	NC

hsa-miR-148a	UCAGUGCACUACAGAACUUUGU	C	C	C
hsa-miR-361	UUAUCAGAAUCUCCAGGGGUAC	C	C	C
hsa-miR-367	AAUUGCACUUUAGCAAUGGUGA	C	C	C
hsa-miR-18a	UAAGGUGCAUCUAGUGCAGAUA	C	C	C
hsa-miR-517a	AUCGUGCAUCCCUUUAGAGUGUU	NC	C	C
hsa-miR-195	UAGCAGCACAGAAAUAUUGGC	C	C	C
hsa-miR-637	ACUGGGGGCUUUCGGGCUCUGCGU	NC	NC	C
hsa-miR-371	GUGCCGCCAUCUUUUGAGUGU	NC	C	C
hsa-miR-183	UAUGGCACUGGUAGAAUUCACUG	C	C	C
hsa-miR-421	AUCAACAGACAUUAAUUGGGCGC	C	C	NC
hsa-miR-517b	UCGUGCAUCCCUUUAGAGUGUU	NC	C	C
hsa-miR-23b	AUCACAUUGCCAGGGAUUACC	C	C	C
hsa-miR-498	UUUCAAGCCAGGGGGCGUUUUUC	NC	C	C
hsa-miR-526a	CUCUAGAGGGAAGCACUUUCU	NC	C	C
hsa-miR-23a	AUCACAUUGCCAGGGAUUUCC	C	C	C
hsa-miR-372	AAAGUGCUGCGACAUUUGAGCGU	NC	C	C
hsa-miR-602	GACACGGGCGACAGCUGCGGCCC	NC	NC	NC
hsa-miR-18b	UAAGGUGCAUCUAGUGCAGUUA	C	C	C
hsa-miR-517*	CCUCUAGAUGGAAGCACUGUCU	NC	NC	NC
hsa-miR-302b*	ACUUUAACAUGGAAGUGCUUUCU	NC	C	C
hsa-miR-335	UCAAGAGCAAUAACGAAAAAUGU	C	C	C
hsa-miR-129	CUUUUUGCGGUCUGGGCUUGC	C	C	C
hsa-miR-19a	UGUGCAAAUCUAUGCAAAACUGA	C	NC	C
hsa-miR-518c*	CAAAGCGCUUCUCUUUAGAGUG	NC	C	C
hsa-miR-524*	CUACAAAGGGAAGCACUUUCUC	NC	C	C
hsa-miR-516-5p	CAUCUGGAGGUAAGAAGCACUUU	NC	NC	C
hsa-miR-185	UGGAGAGAAAGGCAGUUC	C	C	C
hsa-miR-658	GGCGGAGGGAAGUAGGUCCGUUGGU	NC	NC	C
hsa-miR-425-5p	AAUGACACGAUCACUCCCGUUGA	C	C	C
hsa-miR-125a	UCCCUGAGACCCUUUAACCUGUG	C	C	C
hsa-miR-128b	UCACAGUGAACCGGUCUCUUUC	C	C	C
hsa-miR-128a	UCACAGUGAACCGGUCUCUUUU	C	C	C
hsa-miR-29a	UAGCACCAUCUGAAAUCGGUU	C	C	NC
hsa-miR-198	GGUCCAGAGGGGAGAUAGG	NC	C	C
hsa-miR-422b	CUGGACUUGGAGUCAGAAGGCC	NC	C	C
hsa-miR-151	ACUAGACUGAAGCUCCUUGAGG	C	C	C
hsa-miR-148b	UCAGUGCAUCACAGAACUUUGU	C	C	C
hsa-miR-28	AAGGAGCUCACAGUCUAUUGAG	C	C	C
hsa-miR-518f*	CUCUAGAGGGAAGCACUUUCUCU	NC	C	C
hsa-miR-30d	UGUAAACAUCCCCGACUGGAAG	C	C	C
hsa-miR-126	UCGUACCGUGAGUAAUAAUGC	C	C	C
hsa-miR-27b	UUCACAGUGGCUAAGUUCUGC	C	C	C
hsa-miR-24	UGGCUCAGUUCAGCAGGAACAG	C	C	C
hsa-miR-187	UCGUGUCUUGUGUUGCAGCCG	C	C	NC
hsa-miR-512-3p	AAGUGCUGUCAUAGCUGAGGUC	NC	NC	C
hsa-miR-492	AGGACCUGCGGGACAAGAUUCUU	NC	C	C
hsa-miR-526b	CUCUUGAGGGAAGCACUUUCUGUU	NC	C	C
hsa-miR-650	AGGAGGCAGCGCUCUCAGGAC	NC	C	C

hsa-miR-101	UACAGUACUGUGAUAACUGAAG	C	C	C
hsa-miR-564	AGGCACGGUGUCAGCAGGC	NC	NC	C
hsa-miR-574	CACGCUCAUGCACACACCCAC	C	NC	NC
hsa-miR-520h	ACAAAGUGCUUCCCUUUAGAGU	NC	C	C
hsa-miR-520g	ACAAAGUGCUUCCCUUUAGAGUGU	NC	C	C
hsa-miR-30a-5p	UGUAAACAUCCUCGACUGGAAG	C	C	C
hsa-miR-193b	AACUGGCCCUCAAAGUCCCGCUUU	C	C	NC
hsa-miR-301	CAGUGCAAUAGUAUUGUCAAAGC	C	C	C
hsa-miR-565	GGCUGGCUCGCGAUGUCUGUUU	NC	NC	NC
hsa-miR-7	UGGAAGACUAGUGAUUUUGUUG	C	C	C
hsa-miR-135a	UAUGGCUUUUUAUUCCUAUGUGA	C	C	C
hsa-miR-422a	CUGGACUUAGGGUCAGAAGGCC	NC	NC	C
hsa-miR-99b	CACCCGUAGAACCGACCUUGCG	C	C	C
hsa-miR-206	UGGAAUGUAAGGAAGUGUGUGG	C	C	C
hsa-miR-365	UAAUGCCCCUAAAAAUCCUUAU	C	C	C
hsa-miR-572	GUCCGCUCGGCGGUGGCCCA	NC	C	C
hsa-miR-505	GUCAACACUUGCUGGUUUCCUC	C	C	C
hsa-miR-146b	UGAGAACUGAAUUCCAUAGGCU	C	NC	C
hsa-miR-517c	AUCGUGCAUCCUUUUAGAGUGU	NC	C	C
hsa-miR-124a	UUAAGGCACGCGGUGAAUGCCA	C	C	C
hsa-miR-27a	UUCACAGUGGCUAAGUUCCGC	C	C	C
hsa-miR-519d	CAAAGUGCCUCCCUUUAGAGUGU	NC	C	C
hsa-miR-324-3p	CCACUGCCCCAGGUGCUGCUGG	C	C	C
hsa-miR-210	CUGUGCGUGUGACAGCGGCUGA	C	C	C
hsa-miR-373*	ACUCAAAAUGGGGGCGCUUUCC	NC	NC	C
hsa-miR-497	CAGCAGCACACUGUGGUUUGU	C	C	C
hsa-miR-636	UGUGCUUGCUCGUCCCGCCCGCAG	NC	C	C
hsa-miR-484	UCAGGCUCAGUCCCCUCCCGAU	C	C	C
hsa-miR-205	UCCUUCAUUCCACCGGAGUCUG	C	C	C
hsa-miR-296	AGGGCCCCCCCUCAAUCCUGU	C	C	C
hsa-miR-186	CAAAGAAUUCUCCUUUUGGGCUU	C	C	C
hsa-miR-519c	AAAGUGCAUCUUUUUAGAGGAU	NC	C	C
hsa-miR-30e-3p	CUUUCAGUCGGAUGUUUACAGC	C	C	C
hsa-miR-15a	UAGCAGCACAUAAUGGUUUGUG	C	C	C
hsa-miR-214	ACAGCAGGCACAGACAGGCAG	C	C	C
hsa-miR-192	CUGACCUAUGAAUUGACAGCC	C	C	C
hsa-miR-331	GCCCCUGGGCCUAUCCUAGAA	C	C	C
hsa-miR-223	UGUCAGUUUGUCAAAUACCCC	C	C	C
hsa-miR-34a	UGGCAGUGUCUUAGCUGGUUGU	C	C	C
hsa-miR-197	UUCACCACCUUCUCCACCCAGC	C	C	C
hsa-miR-520d	AAAGUGCUUCUCUUUGGUGGGUU	NC	C	C
hsa-miR-601	UGGUCUAGGAUUGUUGGAGGAG	NC	C	C
hsa-miR-598	UACGUCAUCGUUGUCAUCGUCA	NC	NC	C
hsa-miR-191*	GCUGCGCUUGGAUUUCGUCCCC	C	C	C
hsa-miR-515-5p	UUCUCCAAAAGAAAGCACUUUCUG	NC	NC	C
hsa-miR-557	GUUUGCACGGGUGGGCCUUGUCU	NC	NC	C
hsa-miR-520c	AAAGUGCUUCCUUUUAGAGGGUU	NC	C	C
hsa-miR-149	UCUGGCUCCGUGUCUUCACUCC	C	C	C

hsa-miR-489	AGUGACAUCACAUAUACGGCAGC	NC	C	NC
hsa-miR-31	GGCAAGAUGCUGGCAUAGCUG	C	C	C
hsa-miR-525	CUCCAGAGGGAUGCACUUUCU	NC	C	C
hsa-miR-18a*	ACUGCCCUAAGUGCUCCUUCU	C	NC	C
hsa-miR-519e*	AAAGUGCCUCCUUUUAGAGUGU	NC	NC	C
hsa-miR-520f	AAGUGCUUCCUUUUAGAGGGUU	NC	NC	C
hsa-miR-593	AGGCACCAGCCAGGCAUUGCUCAGC	NC	NC	NC
hsa-miR-125b	UCCCUGAGACCCUAACUUGUGA	C	C	C
hsa-miR-204	UUCCCUUUGUCAUCCUAUGCCU	C	C	C
hsa-miR-623	AUCCCUUGCAGGGGCUGUUGGGU	NC	C	C
hsa-miR-519b	AAAGUGCAUCCUUUUAGAGGUUU	NC	C	C
hsa-miR-628	UCUAGUAAGAGUGGCAGUCG	C	C	C
hsa-miR-22	AAGCUGCCAGUUGAAGAACUGU	C	NC	C
hsa-miR-194	UGUAACAGCAACUCCAUGUGGA	C	C	C
hsa-miR-199a*	UACAGUAGUCUGCACAUUGGUU	C	C	C
hsa-miR-340	UCCGUCUCAGUUACUUUAUAGCC	C	C	C
hsa-miR-520a*	AAAGUGCUUCCCUUUGGACUGU	NC	NC	NC
hsa-miR-615	UCCGAGCCUGGGUCUCCCUCU	C	C	C
hsa-miR-200a	UAACACUGUCUGGUAACGAUGU	C	C	C
hsa-miR-155	UUAAUGCUAAUCGUGAUAGGGG	C	C	C
hsa-miR-660	UACCCAUUGCAUAUCGGAGUUG	C	C	C
hsa-miR-181b	AACAUUCAUUGCUGUCGGUGGG	C	C	C
hsa-miR-518b	CAAAGCGCUCCCCUUUAGAGGU	NC	C	C
hsa-miR-532	CAUGCCUUGAGUGUAGGACCGU	C	C	C
hsa-miR-30e-5p	UGUAAACAUCCUUGACUGGA	C	C	C
hsa-miR-560	GCGUGCGCCGGCCGGCCGCC	NC	NC	C
hsa-miR-362	AAUCCUUGGAACCUAGGUGUGAGU	C	C	C
hsa-miR-425-3p	AUCGGGAAUGUCGUGUCCGCC	C	C	C
hsa-miR-9*	UAAAGCUAGAUAACCGAAAGU	C	C	C
hsa-miR-625	AGGGGGAAAGUUCUAUAGUCCU	NC	NC	C
hsa-miR-518f	AAAGCGCUUCUCUUUAGAGGA	NC	NC	C
hsa-miR-429	UAAUACUGUCUGGUAAAACCGU	NC	C	C
hsa-miR-141	UAACACUGUCUGGUAAAGAUGG	C	C	C
hsa-miR-345	UGCUGACUCCUAGUCCAGGGC	NC	C	C
hsa-miR-424	CAGCAGCAAUUCAUGUUUUGAA	C	C	C
hsa-miR-150	UCUCCCAACCCUUGUACCAGUG	C	C	C
hsa-miR-522	AAAAUGGUUCCCUUUAGAGUGUU	NC	C	C
hsa-miR-224	CAAGUCACUAGUGGUUCCGUUUA	C	C	C
hsa-miR-520b	AAAGUGCUUCCUUUUAGAGGG	NC	C	C
hsa-miR-9	UCUUUGGUUAUCUAGCUGUAUGA	C	C	C
hsa-miR-202	AGAGGUAUAGGGCAUGGGAAAA	NC	NC	C
hsa-miR-188	CAUCCCUUGCAUGGUGGAGGGU	C	C	C
hsa-miR-200a*	CAUCUUACCGGACAGUGCUGGA	C	C	C
hsa-miR-518e	AAAGCGCUUCCCUUCAGAGUGU	NC	NC	C
hsa-miR-142-5p	CAUAAAGUAGAAAGCACUAC	C	C	C
hsa-miR-423	AGCUCGGUCUGAGGCCCCUCAG	C	C	C
hsa-miR-181a	AACAUUCAACGCUGUCGGUGAGU	C	C	C
hsa-miR-526c	CUCUAGAGGGAAGCGCUUUCUGUU	NC	C	C

hsa-miR-17-3p	ACUGCAGUGAAGGCACUUGU	C	NC	C
hsa-miR-181d	AACAUUCAUUGUUGUCGGUGGGUU	C	C	C
hsa-miR-563	AGGUUGACAUACGUUUCCC	NC	NC	C
hsa-miR-629	GUUCUCCCAACGUAAGCCCAGC	NC	NC	NC
hsa-miR-203	GUGAAAUGUUUAGGACCACUAG	C	C	C
hsa-miR-589	UCAGAACAAAUGCCGGUUCCCAGA	NC	NC	C
hsa-miR-452	UGUUUGCAGAGGAAACUGAGAC	C	C	C
hsa-miR-654	UGGUGGGCCGCAGAACAUGUGC	NC	C	C
hsa-miR-145	GUCCAGUUUUCCCAGGAAUCCCUU	C	C	C
hsa-miR-526b*	AAAGUGCUUCCUUUUAGAGGC	NC	C	C
hsa-miR-324-5p	CGCAUCCCCUAGGGCAUUGGUGU	C	C	C
hsa-miR-146a	UGAGAACUGAAUUCCAUGGGUU	C	C	C
hsa-miR-652	AAUGGCGCCACUAGGGUUGUGCA	C	C	C
hsa-miR-515-3p	GAGUGCCUUCUUUUGGAGCGU	NC	NC	C
hsa-miR-519a	AAAGUGCAUCCUUUUAGAGUGUUAC	NC	NC	C
hsa-miR-455	UAUGUGCCUUUGGACUACAUCG	C	C	C
hsa-miR-105	UCAAAUGCUCAGACUCCUGU	C	C	C
hsa-miR-143	UGAGAUGAAGCACUGUAGCUCA	C	C	C
hsa-miR-486	UCCUGUACUGAGCUGCCCCGAG	C	C	C
hsa-miR-542-5p	UCGGGGAUCAUCAUGUCACGAG	C	C	NC
hsa-miR-363*	CGGGUGGAUCACGAUGCAAUUU	C	C	NC
hsa-miR-132	UAACAGUCUACAGCCAUGGUCG	C	C	NC
hsa-miR-152	UCAGUGCAUGACAGAACUUGGG	C	C	C
hsa-miR-500	AUGCACCUGGGCAAGGAUUCUG	NC	C	C
hsa-miR-122a	UGGAGUGUGACAAUGGUGUUUGU	C	C	C
hsa-miR-659	CUUGGUUCAGGGAGGGUCCCCA	NC	NC	C
hsa-miR-451	AAACCGUUACCAUUACUGAGUUU	C	C	C
hsa-miR-30a-3p	CUUUCAGUCGGAUGUUUGCAGC	C	C	C
hsa-miR-590	GAGCUUAUUCAUAAAAGUGCAG	NC	C	C
hsa-miR-346	UGUCUGCCCGCAUGCCUGCCUCU	C	C	C
hsa-miR-512-5p	CACUCAGCCUUGAGGGCACUUUC	NC	NC	C
hsa-miR-641	AAAGACAUAGGAUAGAGUCACCUC	NC	NC	C
hsa-miR-330	GCAAAGCACACGGCCUGCAGAGA	C	C	C
hsa-miR-502	AUCCUUGCUAUCUGGGUGCUA	C	C	C
hsa-miR-96	UUUGGCACUAGCACAUUUUUGC	C	C	C
hsa-miR-634	AACCAGCACCCCAACUUUGGAC	NC	NC	C
hsa-miR-215	AUGACCUAUGAAUUGACAGAC	C	C	C
hsa-miR-133b	UUGGUCCCCUUCAACCAGCUA	C	C	C
hsa-miR-501	AAUCCUUUGUCCCUGGGUGAGA	NC	C	C
hsa-miR-550	UGUCUUACUCCCUCAGGCACAU	NC	C	C
hsa-miR-519e	AAAGUGCCUCCUUUUAGAGUGU	NC	NC	C
hsa-miR-218	UUGUGCUUGAUCUAACCAUGU	C	C	C
hsa-miR-212	UAACAGUCUCCAGUCACGGCC	C	C	NC
hsa-miR-133a	UUGGUCCCCUUCAACCAGCUGU	C	C	C
hsa-miR-503	UAGCAGCGGGAACAGUUCUGCAG	C	C	C
hsa-miR-378	CUCCUGACUCCAGGUCCUGUGU	C	C	C
hsa-miR-382	GAAGUUGUUCGUGGUGGAUUCG	C	C	C
hsa-miR-520e	AAAGUGCUUCCUUUUUGAGGG	C	NC	NC

hsa-miR-592	UUGUGUCAAUAUGCGAUGAUGU	C	C	C
hsa-miR-631	AGACCUGGCCCAGACCUCAGC	NC	C	C
hsa-miR-527	CUGCAAAGGGAAGCCCUUUCU	NC	NC	C
hsa-miR-450	UUUUUGCGAUGUGUUCCUAAUA	C	C	C
hsa-miR-127	UCGGAUCCGUCUGAGCUUGGCU	C	C	C
hsa-miR-495	AAACAAACAUGGUGCACUUCUUU	C	C	C
hsa-miR-100	AACCCGUAGAUCCGAACUUGUG	C	C	C
hsa-miR-584	UUAUGGUUUGCCUGGGACUGAG	NC	C	C
hsa-miR-409-3p	CGAAUGUUGCUCGGUGAACCCCU	C	C	C
hsa-miR-504	AGACCCUGGUCUGCACUCUAU	C	C	C
hsa-miR-647	GUGGCUGCACUCACUUCCUUC	NC	NC	NC
hsa-miR-328	CUGGCCCUCUCUGCCCUUCCGU	C	NC	C
hsa-miR-29c	UAGCACCAUUUGAAAUCGGU	C	C	C
hsa-miR-617	AGACUUCCCAUUUGAAGGUGGC	NC	NC	C
hsa-miR-370	GCCUGCUGGGGUGGAACCUGG	C	C	C
hsa-miR-624	UAGUACCAGUACCUUGUGUUCA	NC	C	C
hsa-miR-524	GAAGGCGCUUCCCUUUGGAGU	NC	NC	C
hsa-miR-608	AGGGGUGGUGUUGGGACAGCUCCGU	NC	NC	NC
hsa-miR-520d*	UCUACAAAGGGAAGCCCUUUCUG	NC	C	C
hsa-miR-453	GAGGUUGUCCGUGGUGAGUUCG	C	C	C
hsa-miR-510	UACUCAGGAGAGUGGCAAUCACA	NC	NC	NC
hsa-miR-551b	GCGACCCAUACUUGGUUUCAG	C	C	C
hsa-miR-199a	CCCAGUGUUCAGACUACCUGUUC	C	C	C
hsa-miR-622	ACAGUCUGCUGAGGUUGGAGC	NC	NC	C
hsa-miR-339	UCCCUGUCCUCCAGGAGCUCA	C	C	C
hsa-miR-448	UUGCAUAUGUAGGAUGUCCCAU	C	C	C
hsa-miR-483	UCACUCCUCUCCUCCCGUCUUCU	NC	NC	NC
hsa-miR-596	AAGCCUGCCCGGCUCCUCGGG	NC	NC	C
hsa-miR-431	UGUCUUGCAGGCCGUCAUGCA	C	C	NC
hsa-miR-211	UUCCCUUUGUCAUCCUUCGCCU	C	C	C
hsa-miR-491	AGUGGGGAACCCUUCCAUGAGGA	C	C	C
hsa-let-7a	UGAGGUAGUAGGUUGUAUAGUU	C	C	C
hsa-let-7f	UGAGGUAGUAGAUUGUAUAGUU	C	C	C
hsa-let-7e	UGAGGUAGGAGGUUGUAUAGU	C	C	C
hsa-let-7b	UGAGGUAGUAGGUUGUGUGGUU	C	C	C
hsa-let-7i	UGAGGUAGUAGUUUGUGCUGU	C	C	NC
hsa-let-7c	UGAGGUAGUAGGUUGUAUGGUU	C	C	C
hsa-let-7d	AGAGGUAGUAGGUUGCAUAGU	C	C	C
hsa-let-7g	UGAGGUAGUAGUUUGUACAGU	C	C	C
