# Supplementary material for: Evolutionary Emergence of microRNAs in Human Embryonic Stem Cells
Source: PLoS One. 2008 Jul 30;3(7):e2820. doi: 10.1371/journal.pone.0002820 (PMC2474702; doi:10.1371/journal.pone.0002820)
Supplement: Table S3 — Differential expression levels and evolutionary conservation of hES cell-specific miRNAs during embryoid body (EB) formation. miRNA expression was analyzed in hES cells and EBs after 14 days of differentiation. miRNA expression is arranged by log2 ratio in hES cells/EBs (EB/ES) for 30 of ∼276 miRNAs whose log2 ratio >1. miRNA conservation in various species was determined as described in Table 1. (0.18 MB PDF) [file pone.0002820.s006.pdf]

Table S3

miRNA	log2(EB14/ ES)	Conserved (4 species)	Concerved (rhesus)	Concerved (chimpanzee)
hsa-miR-654	4.95	NC	C	C
hsa-miR-513	3.90	NC	C	C
hsa-miR-494	3.56	C	C	C
hsa-miR-198	3.14	NC	NC	C
hsa-miR-574	2.58	C	NC	NC
hsa-miR-188	2.24	C	C	C
hsa-miR-572	2.22	NC	C	C
hsa-miR-658	1.98	NC	NC	C
hsa-miR-206	1.96	C	C	C
hsa-miR-223	1.89	C	C	C
hsa-miR-601	1.86	NC	C	C
hsa-miR-563	1.74	NC	NC	C
hsa-miR-346	1.63	C	C	C
hsa-miR-498	1.50	NC	C	C
hsa-miR-373*	1.46	NC	NC	C
hsa-miR-373	1.27	NC	C	C
hsa-miR-7	1.14	C	C	C
hsa-miR-575	1.13	NC	NC	C
hsa-miR-602	1.12	NC	NC	NC
hsa-miR-612	1.09	NC	NC	C
hsa-miR-371	1.06	NC	C	C
hsa-miR-367	1.01	C	C	C
hsa-miR-27b	-1.04	C	C	C
hsa-miR-18b	-1.12	C	C	C
hsa-miR-18a	-1.13	C	C	C
hsa-miR-125a	-1.31	C	C	C
hsa-miR-181b	-1.53	C	C	C
hsa-miR-31	-1.65	C	C	C
hsa-miR-594	-1.85	NC	NC	C
hsa-let-7a	-1.87	C	C	C
